# Cell Type‐Specific Modulation of Acute Itch Processing in the Anterior Cingulate Cortex

**DOI:** 10.1002/advs.202403445

**Published:** 2024-09-24

**Authors:** Jiaqi Li, Yang Bai, Junye Ge, Yiwen Zhang, Qiuying Zhao, Dangchao Li, Baolin Guo, Shasha Gao, Yuanyuan Zhu, Guohong Cai, Xiangdong Wan, Jing Huang, Shengxi Wu

**Affiliations:** ^1^ Department of Neurobiology Basic Medical Science Academy Fourth Military Medical University Xi'an 710032 China; ^2^ School of Life Science and Technology ShanghaiTech University Shanghai 201210 China; ^3^ Department of Neurosurgery General Hospital of Northern Theater Command Shenyang 110015 China

**Keywords:** anterior cingulate cortex, GABAergic neurons, glutamatergic neurons, itch, mediodorsal thalamus

## Abstract

Despite remarkable progress in understanding the fundamental bases of itching, its cortical mechanisms remain poorly understood. Herein, the causal contributions of defined anterior cingulate cortex (ACC) neuronal populations to acute itch modulation in mice are established. Using cell type‐specific manipulations, the opposing functions of ACC glutamatergic and GABAergic neurons in regulating acute itching are demonstrated. Photometry studies indicated that ACC glutamatergic neurons are activated during scratching induced by both histamine and chloroquine, whereas the activation pattern of GABAergic neurons is complicated by GABAergic subpopulations and acute itch modalities. By combining cell type‐ and projection‐specific techniques, a thalamocortical circuit is further identified from the mediodorsal thalamus driving the itch‐scratching cycle related to histaminergic and non‐histaminergic itching, which is contingent on the activation of postsynaptic parvalbumin‐expressing neurons in the ACC. These findings reveal a cellular and circuit signature of ACC neurons orchestrating behavioral responses to itching and may provide insights into therapies for itch‐related diseases.

## Graphical abstract

This study identified a thalamocortical circuit from the mediodorsal thalamus driving the itch‐scratching cycle related to histaminergic and non‐histaminergic itching, which is contingent on the activation of postsynaptic parvalbumin‐expressing neurons in the ACC. These findings reveal a cellular and circuit signature of ACC neurons orchestrating behavioral responses to itching and may provide insights into therapies for itch‐related diseases.

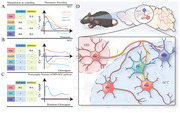

## 1 Introduction

Itching is an aversive sensation that triggers a desire to scratch. Physiological itch protects the body as a defense mechanism, whereas chronic itch disorders associated with dermatosis and systemic diseases take a heavy toll on individual well‐being.^[^ ^1^ ^]^ Based on the release of histamine, chemical itching can be classified into histaminergic and non‐histaminergic subtypes. Histamine activates histamine receptors on the peripheral afferents of sensory neurons, whereas chloroquine (CQ), a canonical non‐histaminergic pruritogen, mainly activates Mas‐related G protein‐coupled receptors.^[^ ^2^, ^3^ ^]^ Subsequently, itching signals travel along primary sensory neurons to the spinal dorsal horn (SDH) and then to the brain via the spinal‐parabrachial/thalamic pathways.^[^ ^4^, ^5^, ^6^ ^]^ Recent studies have revealed the supraspinal circuitry of itch processing, indicating that the central amygdala,^[^ ^7^, ^8^ ^]^ periaqueductal gray (PAG),^[^ ^9^, ^10^ ^]^ and ventral tegmental areas^[^ ^11^, ^12^ ^]^ are involved in itch modulation. However, the cortical mechanisms underlying the itch‐scratching cycle remain elusive.

Human imaging studies have revealed changes in anterior cingulate cortex (ACC) activity during acute itch processing.^[^ ^13^, ^14^, ^15^, ^16^ ^]^Preclinical evidence has shown that chemogenetic or pharmacological inhibition of ACC neurons suppresses histamine‐induced scratching behaviors in rodents, providing direct evidence of the involvement of the ACC in itch modulation.^[^ ^17^, ^18^ ^]^Neurocircuitry studies have further demonstrated that neural projections from the ACC to the dorsal medial stratum (DMS) and from the anteromedial thalamic nucleus (AM) to the ACC selectively facilitate histaminergic itch in mice.^[^ ^19^, ^20^ ^]^The ACC represents a pivotal hub for affective pain processing at the cortical level and exerts endogenous pain facilitation in a top‐down manner.^[^ ^21^, ^22^ ^]^Given the functional heterogeneity of ACC neurons, two separate teams explored the functions of diverse ACC neuronal subpopulations and revealed that ACC glutamatergic neurons, also known as pyramidal neurons (PYN), are effectors of pain sensation and negative emotions, which are negatively regulated by parvalbumin (PV)‐expressing GABAergic interneurons (INs) rather than somatostatin (SST) INs.^[^ ^23^, ^24^ ^]^Meda et al. further demonstrated that activity in specific ACC input‐output pathways, rather than the overall level of ACC activity, underlies the contribution of the ACC to the pain‐related aversion.^[^ ^25^ ^]^However, despite the known heterogeneity of cingulate neurons and circuitry in pain modulation, studies on itch have been limited to unidentified ACC neuronal subpopulations.

To address these questions, we used cell type‐specific optogenetic and chemogenetic tools to manipulate the activity of PYNs and two major IN subtypes in the ACC during acute itch stimulation. Behavioral data indicate contrasting roles of glutamatergic and GABAergic neurons in mediating the sensory aspect of itching. Cell type‐specific calcium imaging recordings revealed that ACC glutamatergic neurons were consistently activated during scratching, whereas excitability changes in GABAergic neurons were complicated by IN subtypes and itch stimuli modality. Using a combination of cell type‐ and projection‐specific techniques, we further identified a thalamocortical circuit originating from the mediodorsal thalamus (MD) that subserves scratching behavior related to both histaminergic and non‐histaminergic itching, which is determined by the recruitment of postsynaptic PV‐expressing neurons in the ACC.

## 2 Results

### 2.1 The ACC is Involved in Acute Itch Processing

**Figure 1:**
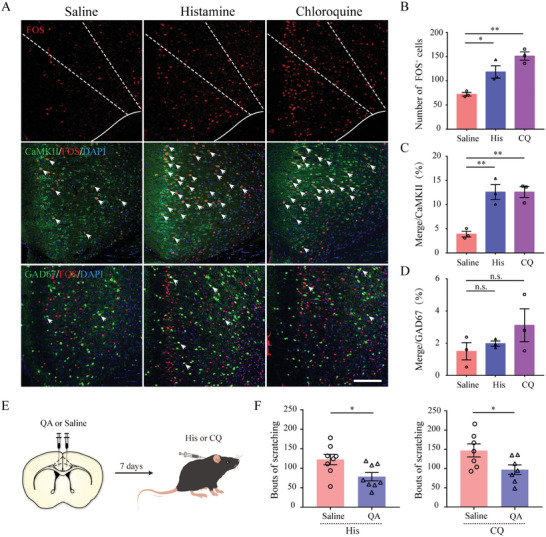
The involvement of the ACC in acute itch processing. A) Representative images of FOS staining in the anterior cingulate cortex (ACC) after saline, histamine, and CQ injection into the nape. Top: single FOS staining in C57BL/6J mice; middle: co‐staining of FOS with CaMKII in C57BL/6J mice; bottom: the expression of FOS in GAD67^+^ neurons in GAD67‐GFP transgenic mice. Arrowheads denote double‐labeled cells. Scale bars: 200 µm. B) Quantification of FOS‐expressing neurons in response to itching agent injection. C–D) Percentages of FOS‐expressing neurons in ACC CaMKII^+^ (C) and GAD67^+^ (D) neurons in response to itching agent injection. n = 3 mice per group, 3 sections per animal. One‐way analysis of variance with post hoc Bonferroni correction test. E) Schematic showing bilateral injection of vehicle or quinolinic acid into the ACC and the experimental timeline for behavioral effect examination. F) Summary qualification of the effect of ACC lesion on scratching induced by histamine (left) and CQ (right). Unpaired t‐test. For histamine stimuli, n = 8 mice per group; for CQ stimuli, n = 7 per group. **P < 0.01, *P < 0.05, ns: not significant.

As the first step to explore the function of the ACC in itch processing, we confirmed using immunohistochemistry that the administration of histamine (100 µg/10 µL) or CQ (100 µg/10 µL) into the nape of the mouse led to a significant increase in FOS‐immunoresponsive neurons in the ACC, suggesting the activation of this cortical area under acute itch conditions (**Figure** **1**A–B). To determine the overall contribution of this cortical area to itch processing, we performed bilateral microinjections of quinolinic acid, a glutamate analog that exerts excitotoxic effects via NMDA receptors (N‐methyl‐D‐aspartic acid receptor), into ACC of C57BL/6J mice (Figure 1E). This resulted in a robust decrease in the number of neurons and an increase in the number of astrocytes in the ACC (Figure S1, Supporting Information). Behavioral experiments showed that ACC lesions, but not vehicle injection, significantly attenuated scratching evoked by histamine and CQ (Figure 1F). These findings imply the role of the ACC in the transmission and regulation of acute itching.

### 2.2 Ca^2+^ Dynamics of ACC Glutamatergic Neurons during Acute Itch

Cortical regions, including the ACC, comprise glutamatergic excitatory projection neurons that transmit signals within and among various nuclei and GABAergic inhibitory INs that control information flow and sculpt network dynamics.^[^ ^21^, ^26^ ^]^ Therefore, we examined the activity of these neuronal subpopulations during itching. Double immunohistochemical labeling was first performed for FOS and CaMKII or GAD67 using a CaMKII antibody or green fluorescent protein (GFP) in GAD67‐GFP mice. Compared to vehicle controls, a higher proportion of CaMKII^+^ neurons, but not GAD67^+^ neurons, expressed FOS in the ACC following injections of both pruritogens (Figure 1A,C,D), suggesting that ACC glutamatergic neurons might primarily be activated by itch stimuli.

**Figure 2:**
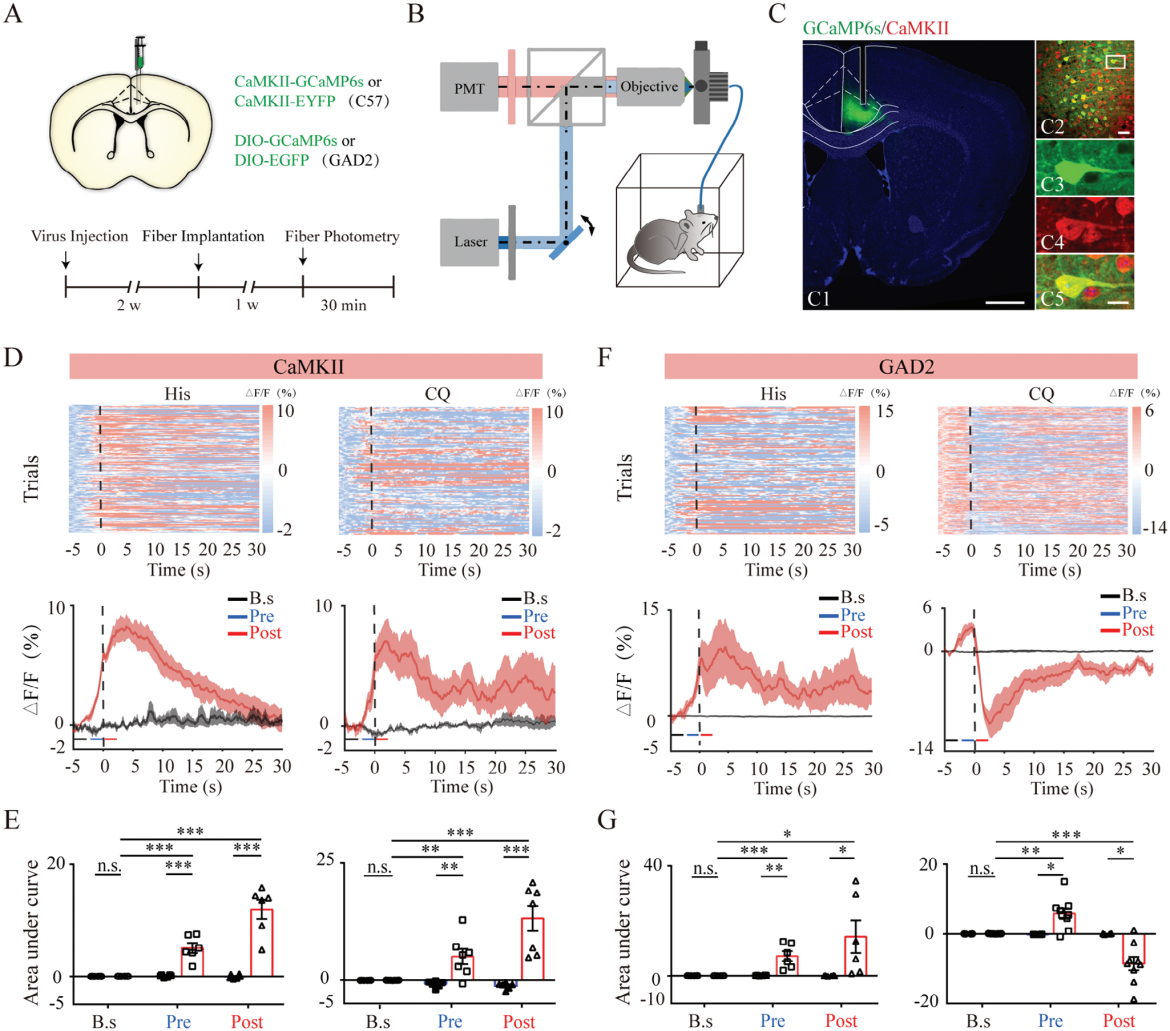
Activity of ACC glutamatergic and GABAergic neurons during acute itch‐induced scratching behavior. A) Schematic showing the viral targeting of CaMKII‐promoted adeno‐associated virus (AAV)‐GCaMP6s into the anterior cingulate cortex (ACC) of C57BL/6J mice and the experimental timeline for Ca^2+^ signal recording using fiber photometry. B) Fiber photometry setup. C) Histological verification of viral expression and optical fiber implantation within the ACC in a representative mouse. The framed area in C2 is magnified in C3–5. Scale bars: 1 mm in (C1), 50 µm in (C2), and 5 µm in (C3–5). D) Ca^2+^ signals recorded from ACC glutamatergic neurons during histamine (left panels) and CQ (right panels) stimuli. Top: Heatmap illustrating the Ca^2+^ signals aligned with the beginning of individual scratching trains in mice injected with AAV‐GCaMP6s. Each row represents Ca^2+^ signals corresponding to one scratching train. The color scale on the right indicates ΔF/F. Bottom: Mean fluorescent signal in response to intradermal injection of itching agents in the nape of mice, with shaded areas indicating the standard error of the mean. The red line denotes the GCaMP6s group, and the black line denotes the EYFP group. The vertical dotted line indicates the scratching bout. E) Area under the curve showing changes in GCaMP6s fluorescence of ACC glutamatergic neurons in the pre‐scratching and post‐scratching periods under the influence of histamine (left) and CQ (right) stimuli. Repeated analysis of variance (ANOVA) followed by simple effects analysis. For histamine stimuli, n = 6 mice in both groups; for CQ stimuli, n = 6 mice in the EYFP group and 7 in the GCaMP6s group. F–G) Recording of ACC GABAergic neurons. The conventions are the same as those in (D–E). Repeated ANOVA followed by simple effects analysis. For histamine stimuli, n = 6 mice in both groups; for CQ stimuli, n = 4 in the EYFP group and n = 9 in the GCaMP6s group. ***P < 0.001, **P < 0.01, * P < 0.05, ns: not significant. PMT, photomultiplier.

Next, we recorded the intracellular Ca^2+^ transients in these subpopulations using in vivo fiber photometry. To visualize glutamatergic neuronal activity related to itch‐induced scratching, we injected recombinant adeno‐associated virus (rAAV) carrying a genetically encoded Ca^2+^ sensor (GCaMP6s) under the control of the CaMKII promoter into the ACC of C57BL/6J mice, with rAAVs containing only a fluorescent tag serving as controls. For sustaining recordings of GCaMP6s fluorescence, we implanted an optical fiber with its tip located within the ACC (**Figure** **2**A–B). GCaMP6s expression in the targeted neurons was verified post hoc by co‐localization with CaMKII (Figure 2C). Three weeks after virus injection, the animals were intradermally injected with histamine (100 µg/10 µL). By aligning GCaMP6s signals in time with video‐recorded scratching trains, we observed that the calcium activity of ACC glutamatergic neurons increased before histamine‐evoked scratching, which lasted at least 20 s after scratching (Figure 2D). To calculate the elevated Ca^2+^ signal across the train of scratching behaviors, we defined the baseline period (−5 to −3 s), pre‐scratching period (−2 to 0 s), and post‐onset period (0–2 s), relative to scratching onset. The results showed that the calcium activity of ACC glutamatergic neurons in the GCaMP6s group was remarkably higher in both the pre‐ and post‐onset periods than its baseline value and the levels found in the control group. The change in the fluorescent signal in these animals was not due to movement artifacts induced by scratching, as the change in EYFP fluorescence was insignificant in control mice (Figure 2E). To extend the above observations to non‐histaminergic itch, we tested the effect of CQ (100 µg/10 µL) injection and obtained similar response patterns (Figure 2D–E). Taken together, these data suggest that ACC glutamatergic neurons exhibit obvious activation that precedes the onset of scratching during acute itching.

### 2.3 Ca^2+^ Dynamics of ACC GABAergic Neurons during Acute Itch

To clarify how acute itch influences GABAergic neurons in the ACC, we infused rAAVs expressing Cre‐dependent GCaMP6s (AAV‐DIO‐GCaMP6s) into unilateral ACC of GAD2‐Cre mice (Figure 2A). Histamine stimulation led to a robust enhancement in GABAergic neuronal activity, which began in the pre‐scratching period and continued for at least 15 s after scratching. However, following nape injection of CQ, GABAergic neurons displayed biphasic responses, with initial increases in the pre‐scratching period and subsequent decreases coinciding with scratching onset in the GCaMP6s fluorescence accompanying each scratching train (Figure 2F–G). Through in vivo calcium imaging, we detected activity changes in GABAergic neurons that were not possible with FOS immunostaining. These data suggest that ACC GABAergic neurons are also involved in central itch processing, although the activation pattern varies with pruritogen type.

**Figure 3:**
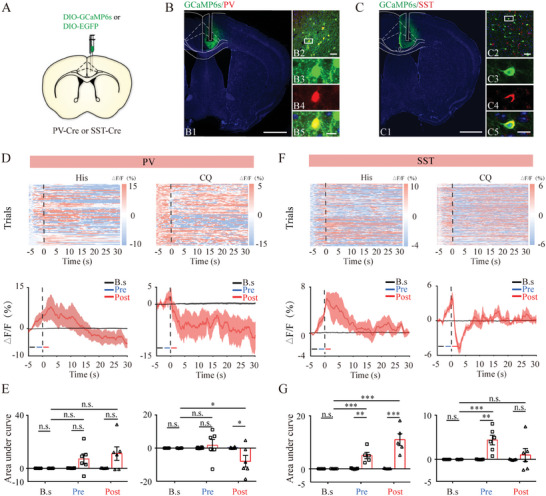
Activity of GABAergic subpopulations during acute itch‐induced scratching behavior. A) Schematic illustration of viral targeting of adeno‐associated virus (AAV)‐DIO‐GCaMP6s into the anterior cingulate cortex (ACC) of parvalbumin (PV)‐Cre or somatostatin (SST)‐Cre mice. B–C) Histological verification of viral expression and optical fiber implantation within the ACC in a representative PV‐Cre (B) or SST‐Cre (C) mouse. The framed area in B2 and C2 is magnified in B3–5 and C3–5. Scale bars: 1 mm (B1, C1), 50 µm (B2, C2), and 5 µm (B3–5, C3–5). D) Ca^2+^ signals recorded from ACC PV‐expressing neurons during histamine (left panels) and CQ (right panels) stimuli. The conventions are the same as those in Figure 2D. E) Area under the curve showing changes in GCaMP6s fluorescence of ACC PV‐expressing neurons in the pre‐scratching and post‐scratching periods under the influence of histamine (left) and CQ (right) stimuli. For histamine stimuli, repeated analysis of variance (ANOVA) and subsequent simple effects analysis, n = 5 mice in the EYFP group and 6 in the GCaMP6s group; for CQ stimuli, repeated ANOVA followed by simple effects analysis, n = 6 in both groups. F–G) Recording of ACC SST‐expressing neurons. The conventions are the same as those in (D–E). Repeated ANOVA followed by simple effects analysis. For histamine stimuli, n = 5 in both groups; for CQ stimuli, n = 5 in the EYFP group and n = 6 in the GCaMP6s group. ***P < 0.001, **P < 0.01, *P < 0.05, NS: not significant.

Next, we focused on neuronal subpopulations expressing either PV or SST, which constitute the majority of ACC GABAergic neurons.^[^ ^26^, ^27^ ^]^ These distinct IN types are differentially modulated by sensory stimuli or motor behavior.^[^ ^28^, ^29^, ^30^ ^]^ We sought to determine the activation dynamics of these two genetically defined inhibitory IN classes in the ACC using fiber photometry, similar to the strategy used in the GABAergic IN experiments (**Figure** **3**A–C). The recordings in PV‐Cre mice (GCaMP6s and control groups) did not reveal any changes in GCaMP6s fluorescent signals before or after histamine treatment‐induced scratching. However, upon CQ stimulation, mice in the GCaMP6s group exhibited significantly decreased fluorescent signals within the ACC compared to baseline values and those in control mice. This change commenced with the onset of scratching and lasted for at least 30 s (Figure 3D–E). Upon recording calcium signals in SST‐Cre mice, we detected a significant increase in fluorescence in both pre‐ and post‐scratching periods upon histamine stimulation compared to the baseline value and in control mice. However, this increase was only observed in the pre‐scratching period after stimulation with CQ (Figure 3F–G). Collectively, ACC PV^+^ neurons were deactivated during the post‐scratching period only upon histamine‐independent itching, whereas ACC SST‐INs were activated with differential temporal patterns in different itch modalities, exhibiting elevated neural activity across the scratching period with histamine stimuli but only in the pre‐scratching period with CQ stimuli.

To rule out the possibility that movement‐related behavior modulates the activity of ACC neuronal subpopulations during acute itch, we analyzed the changes in fluorescence values across the trains of locomotion during CQ stimuli in the C57BL/6J and transgenic mice lines mentioned above that received AAV‐GCaMP6s injection in the ACC. Six mice in the four lines of mice for Ca^2+^ dynamic monitoring mentioned above were included for further analysis. Overall, locomotion including digging, grooming, walking, and standing did not influence the activity of these neuronal subpopulations (Figure S2, Supporting Information). These data highlight the specificity of ACC neuronal activity to scratching under the condition of acute itch stimuli.

### 2.4 Behavioral Effects of Manipulating ACC Glutamatergic Neurons during Acute Itching

**Figure 4:**
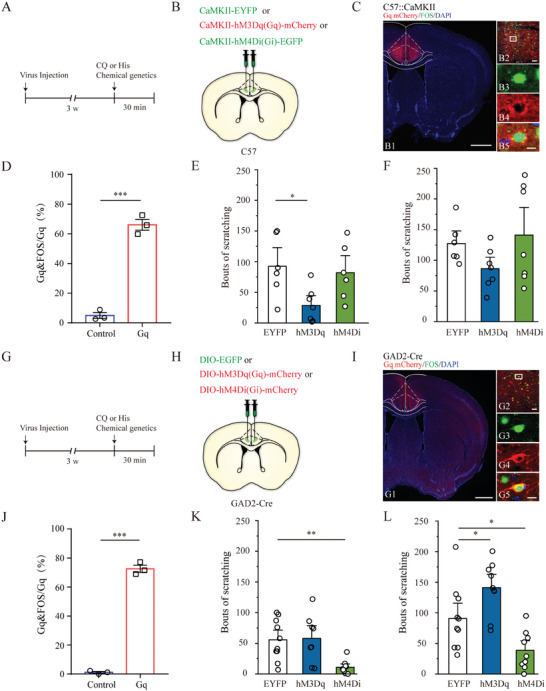
Effects of chemogenetic manipulation of ACC glutamatergic and GABAergic neurons on acute itch‐induced scratching. A‐B) Timeline of experimental design and schematic illustration of viral injection for chemogenetic modulation of anterior cingulate cortex (ACC) glutamatergic neurons. C) Histological verification of viral expression within the ACC in a representative mouse injected with adeno‐associated virus (AAV)‐CaMKII‐hM3Dq‐mCherry (left) and FOS expression (green) in a representative mCherry‐labeled neuron (red) in response to clozapine N‐oxide (CNO) administration (right). The framed area in B2 is magnified in B3–5. D) CNO administration increased the expression of FOS in mCherry‐labeled neurons in mice injected with AAV‐CaMKII‐hM3Dq‐mCherry compared to that in control mice. N = 3 mice per group, 3 sections per animal. Unpaired *t*‐test: *t* = 14.567, df = 4. E) Chemogenetic activation of ACC glutamatergic neurons reduces scratching behavior induced by histamine. One‐way ANOVA followed by Bonferroni correction test. N = 6 mice in EYFP and hM4Di groups, and 7 in the hM3Dq group. F) Chemogenetic activation or inactivation of ACC glutamatergic neurons does not affect scratching behavior induced by CQ. Kruskal‐Wallis H test. N = 6 mice in the EYFP group and 7 in the hM3Dq and hM4Di groups. G‐H) Schematic illustration of viral injection for chemogenetic modulation of ACC GABAergic neurons. I) Histological verification of viral expression within the ACC of a representative GAD2‐Cre mouse injected with AAV‐DIO‐hM3Dq‐mCherry (left), and FOS expression (green) in a representative mCherry‐labeled neuron (red) in response to CNO administration (right). The framed area in G2 is magnified in G3–5. J) CNO administration increases the expression of FOS in mCherry‐labeled neurons in mice injected with AAV‐DIO‐hM3Dq‐mCherry compared to control mice. N = 3 mice per group, 3 sections per animal. Unpaired *t*‐test: *t* = 28.472, df = 4. K) Chemogenetic inactivation of ACC GABAergic neurons decreases scratching behaviors induced by histamine. Kruskal‐Wallis H test. N = 10 mice in the EYFP group, 8 in the hM3Dq group, and 9 in the hM4Di group. L) Chemogenetic activation of ACC GABAergic neurons increases scratching behavior induced by CQ, whereas chemogenetic inhibition of them reduces scratching behavior. One‐way ANOVA followed by Bonferroni correction test. N = 10 mice in the EYFP group and 8 in the hM3Dq and hM4Di groups. ***P < 0.001, **P < 0.01, *P < 0.05, NS: not significant.

Our fiber photometry data suggest a potential role for cingulate glutamatergic and GABAergic neurons in itch processing. Prior data have shown that bidirectional manipulation of these neuron subtypes via optogenetics or chemogenetics did not interfere locomotion in the control mice,^[^ ^23^, ^31^ ^]^ laying the basis for our explorations for the causal relationships between ACC activity manipulation and acute itch‐related scratching behavior with the same methods. A pharmacogenetic approach using Designer Receptors Exclusively Activated by Designer Drugs (DREADDs) was first used to manipulate specific ACC neuronal subpopulations. We first targeted glutamatergic neurons by locally injecting rAAVs into bilateral ACC of C57BL/6J mice, which delivered a construct containing the CaMKIIα promoter‐driven excitatory DREADD hM3Dq or inhibitory hM4Di fused with mCherry. Mice injected with rAAVs containing only a fluorescent tag served as controls (**Figure** **4**A–B). The efficiency of DREADDs was verified using FOS staining of ACC slices from mice injected with hM3Dq and a greater number of FOS‐expressing neurons were observed in the hM3Dq group than in control mice after intraperitoneal administration of the exogenous DREADD ligand clozapine N‐oxide (CNO) (Figure 4C‐D). Behavioral data showed that pharmacogenetic activation of these neurons greatly attenuated scratching behavior only evoked by histamine (Figure 4E), despite a trend toward decreased number of scratching seen upon CQ stimuli (Figure 4F); whereas their inhibition did not affect the number of scratches induced by histamine or CQ injection (Figure 4E‐F). In addition, pharmacogenetic modulation of ACC glutamatergic neurons did not elicit spontaneous scratching bouts in naïve mice (Figure S3A‐B, Supporting Information).

**Figure 5:**
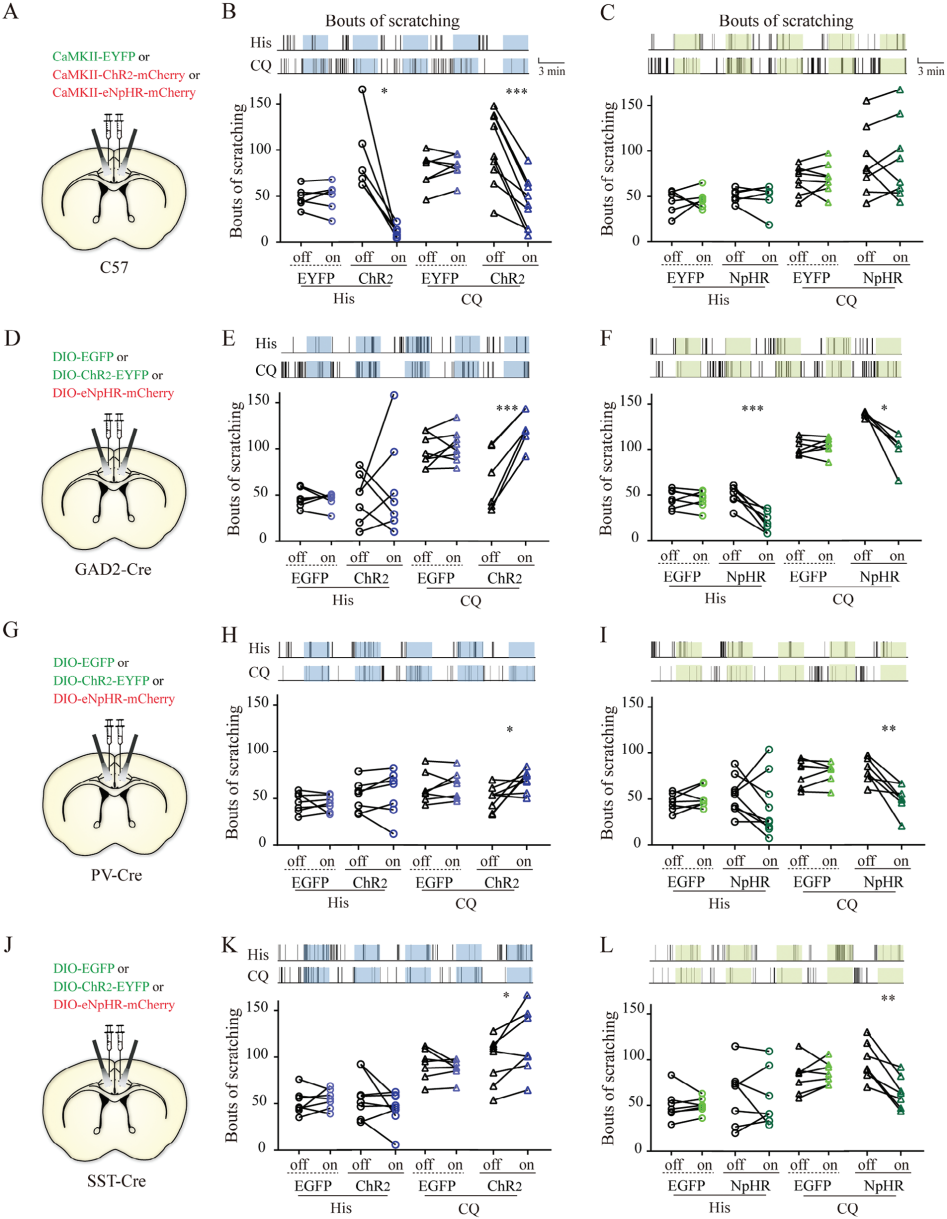
Effects of optogenetic manipulation of ACC glutamatergic and GABAergic neurons on acute itch‐induced scratching. A) Schematic illustration of viral injection for optical modulation of anterior cingulate cortex (ACC) glutamatergic neurons. B) Optical activation of ACC glutamatergic neurons reduces scratching behaviors induced by histamine and CQ. Top: Representative raster plots illustrating scratching episodes in mice with AAV‐CaMKII‐ChR2 injection following blue light illumination. For histamine stimuli, paired t‐test for the EYFP group, n = 6 mice; Wilcoxon signed‐rank test for the ChR2 group, n = 6. For CQ stimuli, paired t‐test for the EYFP group, n = 7; paired t‐test for the ChR2 group, n = 9. C) Optical inactivation of ACC glutamatergic neurons exerts no effect on acute itch‐evoked scratching behaviors. For histamine stimuli, paired t‐test for the EYFP group, n = 6; Wilcoxon signed‐rank test for the NpHR group, n = 6. For CQ stimuli, paired t‐test, n = 8 in both groups. D) Schematic illustration of viral injection for optical modulation of ACC GABAergic neurons. E) Optical activation of ACC GABAergic neurons increases scratching behaviors induced by CQ. For histamine stimuli, Wilcoxon signed‐rank test for the EYFP group, n = 7; paired t‐test for the ChR2 group, n = 7. For CQ stimuli, paired t‐test, n = 8 in the EYFP group and n = 6 in the ChR2 group. F) Optical inactivation of ACC GABAergic neurons reduces scratching behaviors induced by histamine and CQ. Paired t‐test. For histamine stimuli, n = 7 in both groups. For CQ stimuli, n = 7 in the EYFP group and n = 5 in the NpHR group. G) Schematic illustration of viral injection for optical modulation of ACC parvalbumin (PV)‐expressing neurons. H) Optical activation of ACC PV‐expressing neurons increases scratching behaviors induced by CQ. Paired t‐test. For histamine stimuli, n = 7 in both groups. For CQ stimuli, n = 7 in the EYFP group and n = 8 in the ChR2 group. I) Optical inactivation of ACC PV‐expressing neurons reduces scratching behaviors induced by histamine and CQ. Paired t‐test. For histamine stimuli, n = 7 in the EYFP group and n = 9 in the NpHR group. For CQ stimuli, n = 7 in both groups. J) Schematic illustration of viral injection for optical modulation of ACC somatostatin (SST)‐expressing neurons. K) Optical activation of ACC SST‐expressing neurons selectively increases scratching behaviors induced by CQ. Paired t‐test. For histamine or CQ stimuli, n = 7 in the EYFP group and n = 9 in the ChR2 group. L) Optical inactivation of ACC SST‐expressing neurons selectively reduces scratching behaviors induced by CQ but not histamine. Paired t‐test. For histamine or CQ stimuli, n = 7 in both groups. The conventions in E–F, H–I, and J–K are the same as in B–C. ***P < 0.001, **P < 0.01, *P < 0.05, NS: not significant.

Compared to chemogenetics, which modulates neuronal firing for several hours with a single dose of CNO, optogenetics excels at providing immediate control of neuronal firing with light pulses and precisely linking neuronal activity to behaviors.^[^ ^32^ ^]^ Thus, to validate the chemogenetic data, we expressed rAAV‐CaMKIIα‐ChR2‐mCherry and rAAV‐CaMKIIα‐eNpHR‐mCherry in bilateral ACC of C57BL/6J mice, followed by the implantation of optical fibers in the middle of the injection site (**Figure** **5**A; Figure S4A–B, Supporting Information). Remarkably, photoactivation of ACC glutamatergic neurons via blue light induced an immediate decrease in scratching behavior induced by both pruritogens, which returned to baseline values when the light was turned off. Compared to the light‐off period, the mice exhibited a significantly decreased number of scratches during the light‐on period under conditions of both histamine‐ and CQ‐induced itching (Figure 5B). However, optogenetic inactivation of ACC glutamatergic neurons by yellow light did not influence the number of scratches when the mice were treated with histamine or CQ (Figure 5C). Consistent with the chemogenetic data, these findings suggest that ACC glutamatergic neurons play a pivotal role in inhibiting the itch‐scratching cycle.

### 2.5 Behavioral Effects of Manipulating ACC GABAergic Neurons during Acute Itching

To establish a causal link between GABAergic neuronal activity in the ACC and acute itch‐induced scratching behavior, we examined the effects of chemogenetic and optogenetic modulation of GABAergic neurons on itch‐induced scratching. For chemogenetics, we stereotaxically injected rAAV encoding Cre‐dependent hM3Dq‐mCherry or hM4Di‐mCherry into bilateral ACC of GAD2‐Cre mice, using rAAV‐DIO‐EGFP as a control (Figure 4G–H). Efficient chemogenetic modulation was confirmed using FOS immunostaining (Figure 4I‐J). Behaviorally, chemogenetic activation of ACC GABAergic neurons significantly enhanced scratching related to CQ, but not histamine, whereas chemogenetic inactivation of these neurons greatly attenuated both histamine‐ and CQ‐evoked scratching behaviors (Figure 4K). As with glutamatergic neurons, ACC GABAergic neurons did not affect spontaneous scratching bouts upon pharmacologic modulation in the absence of acute itch stimuli (Figure S3C‐D). For optogenetics, we infused AAV encoding Cre‐dependent ChR2‐EYFP and eNpHR‐mCherry into the ACC of GAD2‐Cre mice and implanted optical fibers as described previously, using rAAV‐hSyn‐DIO‐EGFP as a control (Figure 5D; Figure S4C–D, Supporting Information). Consistent with our chemogenetic experimental data, blue light‐induced activation of these neurons significantly enhanced scratching related to CQ but not histamine (Figure 5E), whereas optical inhibition significantly suppressed both histamine‐ and CQ‐induced itching (Figure 5F). Taken together, GABAergic neurons in the ACC positively regulate scratching behavior during acute itching.

Different types of ACC INs exhibit diverse behavioral correlations, including sensory processing.^[^ ^23^, ^33^ ^]^ To link the identified GABAergic neuronal types with itch behaviors, we next sought to determine the role of PV^+^ or SST^+^ INs in acute itch processing using optogenetics, similar to the strategy used in the GABAergic neuron experiments in PV‐ and SST‐Cre mice (Figure 5G, J; Figure S4E–H, Supporting Information). Behaviorally, neither optical activation nor inactivation of PV‐INs significantly affected histamine‐dependent itching. However, in mice subjected to CQ stimuli, photoactivation of PV‐INs resulted in a significant increase in scratching behavior, and optogenetic deactivation induced a robust decrease in scratching behavior compared to pre‐stimulation baseline values and those in photostimulated control mice (Figure 5H–I). In addition, we observed no behavioral changes in histamine‐induced scratching with upregulation or downregulation of SST neuronal activity. CQ‐evoked scratching was potentiated by the optical activation of SST‐INs but suppressed by their inactivation (Figure 5K–L). These results are consistent with those observed when PV‐INs are modulated by optogenetics. In summary, both PV^+^ and SST^+^ INs facilitate histamine‐independent itch processing.

### 2.6 Effects of Activating GABA Interneuron Subtypes on ACC Network Activity

**Figure 6:**
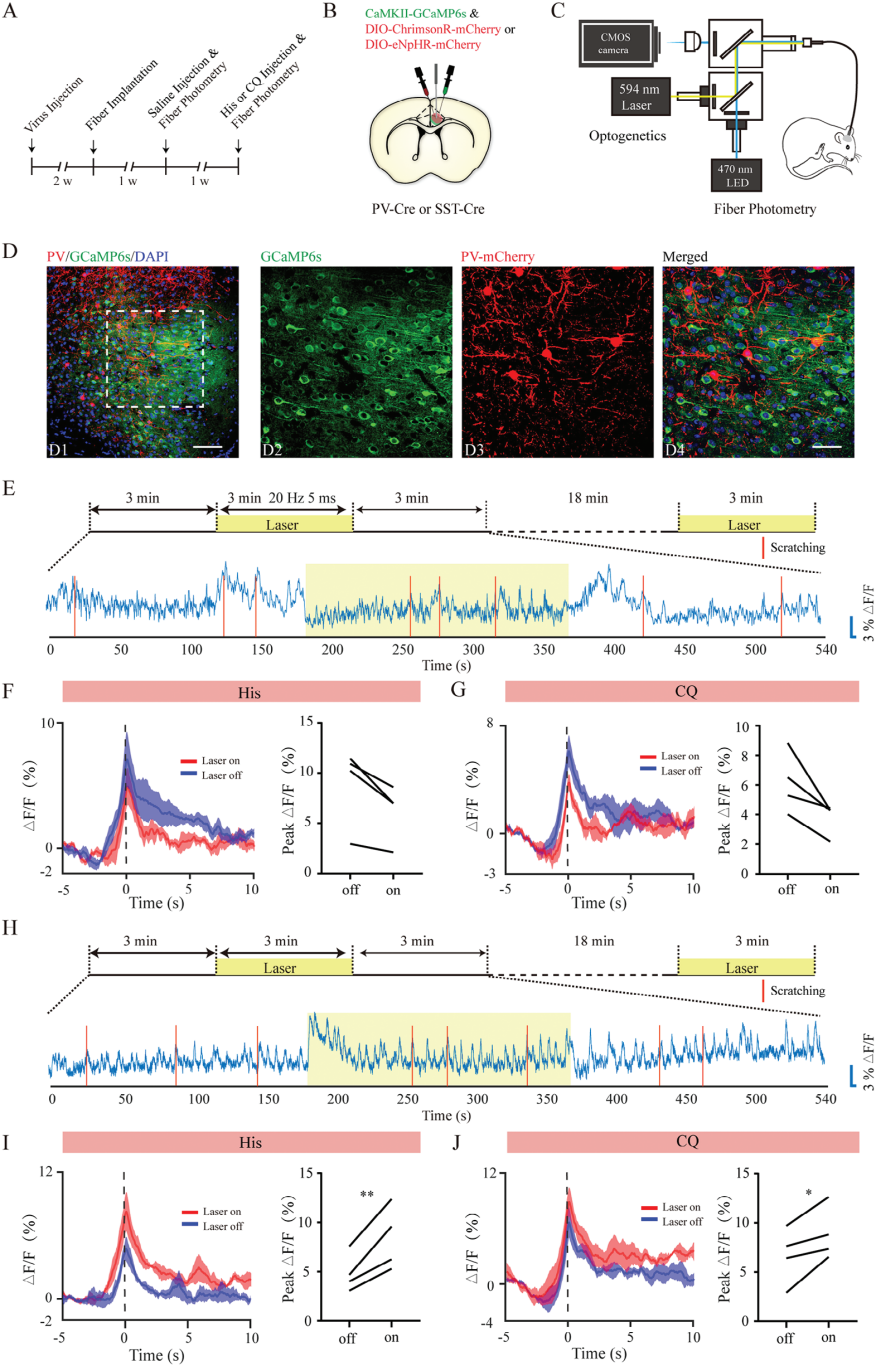
Effects of optogenetic manipulation of ACC PV‐expressing neurons on the activity of glutamatergic neurons during acute itch. A) Timeline of experimental design. B–C) Schematic illustration of virus injection (B) and fiber photometry setup (C) to achieve simultaneous in vivo photometry recording and optogenetic manipulation. D) A representative photograph showing CaMKII‐GCaMP6s labeled glutamatergic neurons (green) and DIO‐ChrimsonR‐mCherry‐labeled parvalbumin (PV) neurons (red) in PV‐Cre mice. Scale bars: 100 µm (D1) and 50 µm (D2–4). E) A sample trace of calcium fluorescence changes of anterior cingulate cortex (ACC) glutamatergic neurons related to scratching induced by histamine stimuli in the light‐on and light‐off periods. (F‐G) The effect of optical activation of PV‐expressing neurons on the activity of ACC glutamatergic neurons during histamine F) and chloroquine G) induced scratching. The conventions are the same as in Figure 2D‐E. Wilcoxon signed‐rank test. N = 4 mice. H–J) The effect of optical inhibition of PV‐expressing neurons on the activity of ACC glutamatergic neurons during acute itch‐induced scratching. The conventions are the same as in (E–G). Paired t‐test. N = 4 mice. **P < 0.01, *P < 0.05.

ACC function depends on a delicate balance between excitation and inhibition, involving various types of monosynaptic connections between PYNs and INs.^[^ ^34^, ^35^ ^]^ Intrigued by the distinct activity patterns of ACC PYNs and GABAergic neurons as well as their opposing effects on acute itch, we investigated how these GABAergic subpopulations affect ACC network activity during acute itching using a combination of optogenetic manipulation and calcium recording.^[^ ^36^ ^]^ Within the ACC of PV‐Cre or SST‐Cre transgenic mice, we co‐expressed two AAVs: one expressing GCaMP6s under the CaMKII promoter and the other expressing Cre‐dependent excitatory opsin ChrimsonR or inhibitory opsin eNpHR. This allowed us to simultaneously track the calcium activity of ACC PYNs at the population level, while optogenetically activating or silencing PV or SST neurons in awake mice (**Figure** **6**A–C). We first characterized the response dynamics of ACC PYNs in mice under basal conditions and found that ACC PYNs showed significant decreases in Ca^2+^ transients upon photoactivation of both PV and SST neurons but showed opposite responses upon photoinhibition (Figure S5, Supporting Information). To explore whether these regulatory effects changed under the condition of acute itch, we repeated the assays in the case of histamine and CQ stimuli, with similar results obtained (Figure 6D–J; Figure S6, Supporting Information). Collectively, these data confirm the inhibitory role of PV and SST neurons in regulating the general ACC network activity, corroborating the identified synaptic connections from INs to PYNs at the single‐cell level via electrophysiological recordings.^[^ ^34^ ^]^

### 2.7 Identification of a Thalamocortical Pathway Involved in Acute Itch Processing

**Figure 7:**
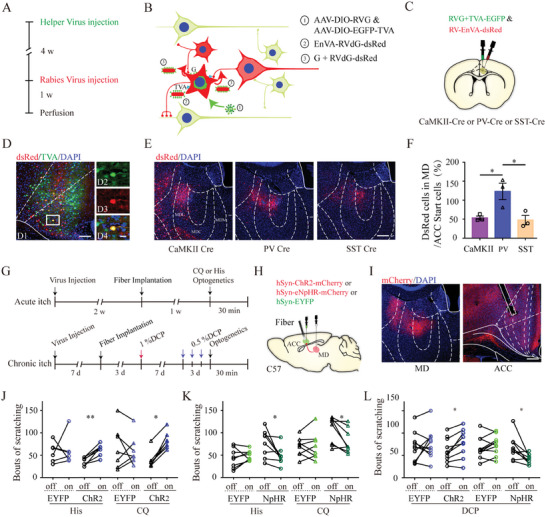
Effects of optogenetic manipulation of the MD‐ACC pathway on acute and chronic itch‐induced scratching. A) Timeline of monosynaptic rabies tracing. B) Schematic of the monosynaptic retrograde tracing strategy. C) Schematic of monosynaptic rabies tracing. D) A representative photograph of a coronal section containing anterior cingulate cortex (ACC) of CaMKII‐Cre mouse showing the expression of adeno‐associated virus (AAV)‐EGFP (green) and rabies virus (RV)‐dsRed (red). Cells co‐expressing AAV‐EGFP and RV‐dsRed are considered starter cells (yellow). Scale bars: 100 µm (D1) and 20 µm (D2–4). E) Representative photographs showing the existence of retrograde labeled cells (red) in the mediodorsal thalamic nucleus (MD) of CaMKII‐Cre, parvalbumin (PV)‐Cre, and somatostatin (SST)‐Cre mice. F) Bar graph showing that more presynaptic neurons per starter cell could be seen in the ACC of PV‐Cre mouse than CaMKII‐Cre and SST‐Cre mice. n = 3 mice per group, 3 sections per animal. One‐way analysis of variance followed by Bonferroni test. G) Timeline of experimental design for optical modulation of the MD‐ACC pathway. H) Schematic of viral injection. I) Representative photographs showing the virus injection site in the mediodorsal thalamic nucleus (left) and mCherry‐labeled fibers and terminals as well as the site of fiber implantation in the ipsilateral anterior cingulate cortex (right). Scale bars: 200 µm. J) Photoactivation of the MD‐ACC pathway increases the amount of scratching induced by histamine and CQ. For histamine stimuli: Wilcoxon signed‐rank test for the EYFP group, n = 6; paired t‐test for the ChR2 group, n = 7. For CQ stimuli: paired t‐test for the EYFP group, n = 7; Wilcoxon signed‐rank test for the ChR2 group, n = 7. K) Photoinhibition of the MD‐ACC pathway decreases the amount of scratching induced by histamine and CQ. For histamine stimuli: paired t‐test for the EYFP group, n = 8; paired t‐test for the ChR2 group, n = 8. For CQ stimuli: paired t‐test for the EYFP group, n = 8; Wilcoxon signed‐rank test for the ChR2 group, n = 8. L) Photoactivation of the MD‐ACC pathway increases the amount of scratching induced by DCP, while photoinhibition of this pathway decreases the amount of scratching. Paired t‐test. For photoactivation: n = 10 in both groups; for photoinhibition: n = 10 in the EYFP group and 9 in the NpHR group. **P < 0.01, *P < 0.05.

Our aforementioned findings elucidate the functional roles of local ACC neuronal subtypes in acute itch processing. Next, we delved into understanding the foundational neurocircuitry input to support the modulatory role of the ACC in chemical itching. Previous neuroanatomical studies based on traditional neural tracers revealed that the ACC integrated inputs from many cortical and thalamic areas but failed to identify brain inputs to a specific type of neuron.^[^ ^37^, ^38^ ^]^ Herein, we employed a rabies‐based, retrograde, transsynaptic tracing approach to identify whole‐brain monosynaptic inputs into glutamatergic, PV‐expressing, and SST‐expressing neurons in the ACC using the corresponding transgenic mouse lines expressing Cre recombinase in these neurons (**Figure** **7**A–C). Starter neurons (EGFP and dsRed double‐labeled neurons) were localized in the ACC (Figure 7D). There was no significant difference in the distribution pattern of the upstream neurons among the three mouse lines, and intense dsRed‐labeled neurons were observed in the AM, MD, retrosplenial cortex, and visual cortex (Figure S7, Supporting Information). However, it is worth to mention that the ratio of rabies virus (RV)‐labeled cells in the MD to ACC starter cells in PV‐Cre mice was much higher than that in CaMKII‐Cre and SST‐Cre mice (Figure 7E–F). These data, in accordance with our recent mammalian GFP reconstitution across synaptic partners data,^[^ ^39^ ^]^ imply that MD neurons preferentially project onto ACC PV‐expressing neurons.

As a key relay for nociceptive information, the thalamus is necessary for sensory processing via thalamocortical pathways.^[^ ^40^ ^]^ Among these thalamic regions, the AM‐ACC pathway promotes histaminergic itch modulation.^[^ ^20^ ^]^ The MD‐ACC inputs drive negative pain‐related affect,^[^ ^25^ ^]^ but their role in itch processing remains elusive. Before manipulating the excitatory MD inputs to the ACC, we first tested the effects of chemogenetic modulation of MD neurons on acute itch and surprisingly found no change in the number of scratching bouts upon MD action or inactivation (Figure S8, Supporting Information). Then, we employed projection‐specific optogenetics by injecting rAAV2/9‐hsyn‐hChR2‐mCherry or rAAV2/9‐hsyn‐eNpHR‐mCherry into the MD with an optical fiber implanted over the ACC in C57BL/6J mice (Figure 7G‐H). Dense mCherry‐labeled fibers and terminals were observed in layers II–III and V of the ACC (Figure 7I). Behavioral assays showed that optogenetically activating MD terminals in the ACC increased scratching behaviors induced by acute chemical itch and allergic contact dermatitis‐related chronic itch, while inhibiting this pathway attenuated scratching evoked by these itch modalities (Figure 7J–L). These results demonstrate that a specific subpopulation of MD neurons, including those projecting to the ACC, participates in acute itch processing.

### 2.8 Microcircuit Mechanisms through which MD Input to ACC Facilitate Acute Chemical Itching

**Figure 8:**
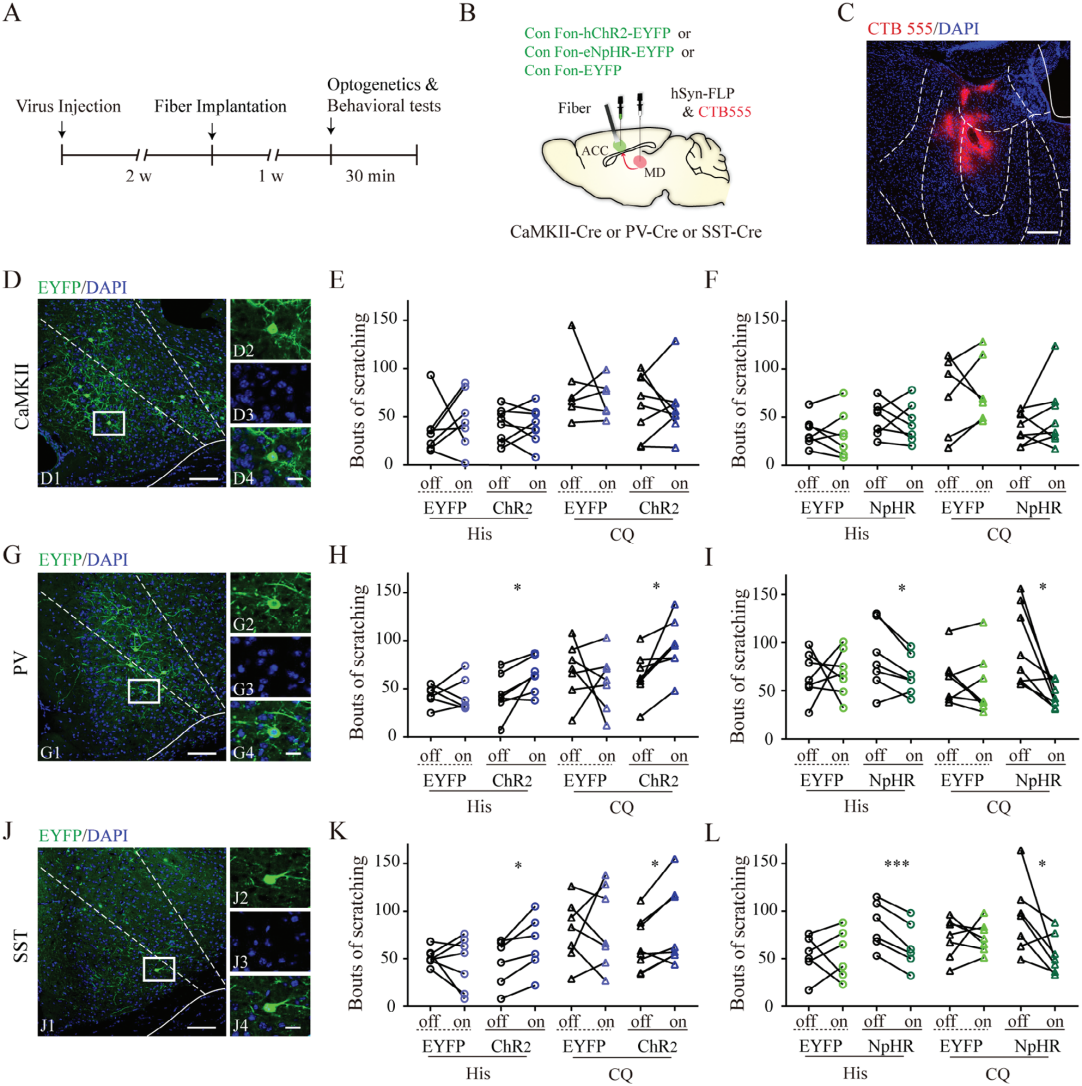
Effects of optogenetic manipulation of postsynaptic glutamatergic, PV‐expressing, and SST‐expressing neurons of the MD‐ACC pathway on acute itch‐induced scratching. A) Timeline of experimental design. B) Schematic illustration of monosynaptic anterograde tracing. C) Histological verification of hsyn‐FLP injection site in the mediodorsal thalamic nucleus with the aid of CTB 555. D) A representative photograph showing anterograde labeled cells (green) in the anterior cingulate cortex (ACC) of CaMKII‐Cre mice. E) Optical activation of postsynaptic glutamatergic neurons exerted no effect on acute itching. For histamine stimuli: Wilcoxon signed‐rank test for the EYFP group, n = 7 mice; paired t‐test for the ChR2 group, n = 8. For CQ stimuli: paired t‐test, n = 6 in the EYFP group and n = 8 in the ChR2 group. F) Optical inactivation of postsynaptic glutamatergic neurons exerted no effect on acute itching. Paired t‐test. For histamine stimuli, n = 7 in both groups. For CQ stimuli, n = 6 in the EYFP group and n = 8 in the NpHR group. G) A representative photograph showing anterograde labeled cells (green) in the ACC of parvalbumin (PV)‐Cre mice. H) Optical activation of postsynaptic PV‐expressing neurons facilitated both histaminergic and non‐histaminergic acute itch. Paired t‐test. For histamine stimuli, n = 6 in the EYFP group and n = 7 in the ChR2 group. For CQ stimuli, n = 7 in both groups. I) Optical inactivation of postsynaptic PV‐expressing neurons suppressed acute itch‐induced scratching. For histamine stimuli: paired t‐test, n = 7 in both groups. For CQ stimuli: Wilcoxon signed‐rank test for the EYFP group, n = 8; paired t‐test for the NpHR group, n = 8. J) A representative photograph showing anterograde labeled cells (green) in the ACC of somatostatin (SST)‐Cre mice. K) Optical activation of postsynaptic SST‐expressing neurons facilitated both histaminergic and non‐histaminergic acute itching. Paired t‐test. For histamine stimuli: n = 7 in the EYFP group and n = 6 in the ChR2 group. For CQ stimuli: n = 7 in the EYFP group and n = 8 in the ChR2 group. L) Optical inactivation of postsynaptic SST‐expressing neurons suppressed acute itch‐induced scratching. Paired t‐test. For histamine stimuli, n = 6 in both groups. For CQ stimuli, n = 7 in both groups. The framed areas in D1, G1, and J1 are magnified in D2–4, G2–4, and J2–4. Scale bars: 100 µm in (C), 1 mm in (D1, G1, and J1), 50 µm in (D2–4, G2–4, and J2–4). ***P < 0.001, *P < 0.05.

We then aimed to determine the role of cingulate postsynaptic neuron subtypes of MD‐ACC projection neurons in itch transmission. To label these neuron subtypes, we infused anterograde trans‐monosynaptic rAAV2/1‐hSyn‐FLP into bilateral MD of CaMKIIα‐Cre, PV‐Cre, and SST‐IRES‐Cre mice, and then injected rAAV‐hSyn‐Con/Fon‐EYFP into bilateral ACC. Under these conditions, the expression of ChR2‐ or eNpHR3.0‐EYFP was dependent on both Cre and FLP recombinases and only cingulate glutamatergic, PV‐expressing, or SST‐expressing neurons that received inputs from MD expressed EYFP. In addition, rAAV‐hSyn‐Con/Fon‐eNpHR3.0‐EYFP or rAAV‐hSyn‐Con/Fon‐hChR2‐EYFP were injected for optical manipulation (**Figure** **8**A–C). We found dense EYFP‐positive neurons in layers II–III of the ACC in all mouse lines, with a few neurons scattered in layer V (Figure 8D,G,J). Surprisingly, photoactivation of the cingulate postsynaptic glutamatergic neurons of the MD‐ACC pathway did not affect histamine‐ or CQ‐induced itch behavior (Figure 8E–F). However, optogenetically activating postsynaptic PV‐ or SST‐expressing neurons increased both histamine‐ and CQ‐induced itch responses, whereas photoinhibition reduced acute itch‐related scratching (Figure 8H–I,K–L). These data suggest that the MD‐ACC^PV^ and MD‐ACC^SST^ pathways, rather than their glutamatergic counterparts, facilitate acute itch processing.

**Figure 9:**
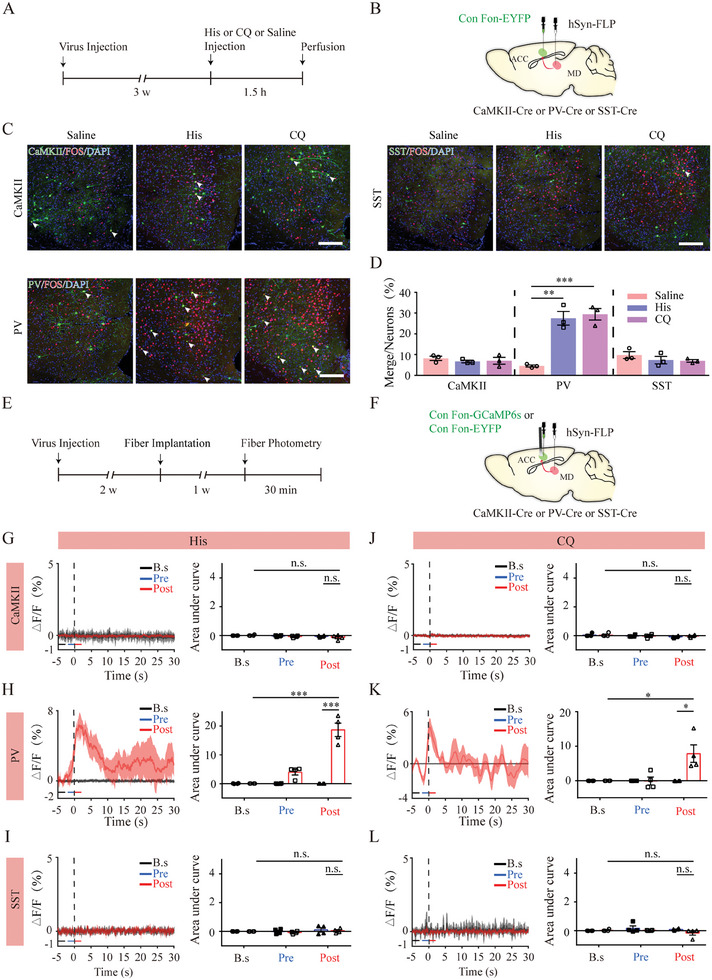
Neural activity changes of postsynaptic glutamatergic, PV‐expressing, and SST‐expressing neurons of the MD‐ACC pathway upon acute itch stimuli. A) Timeline of experimental design for FOS immunostaining. B) Schematic illustration of monosynaptic anterograde tracing. C) Representative photographs showing the expression of FOS (red) in anterograde labeled cells (green) in the anterior cingulate cortex (ACC) of CaMKII‐Cre, parvalbumin (PV)‐Cre, and somatostatin (SST)‐Cre mice. Scale bars: 200 µm. D) Bar graph showing that acute histaminergic and non‐histaminergic itch stimulate FOS expression in postsynaptic PV‐expressing neurons instead of glutamatergic or SST‐expressing neurons. n = 3 mice per group, 3 sections per animal. One‐way analysis of variance (ANOVA) followed by Bonferroni test. E) Timeline of experimental design for calcium recordings. F) Schematic illustration of monosynaptic anterograde tracing and calcium recordings. G–I) Ca^2+^ signals recorded from ACC glutamatergic G), PV‐expressing (H), and SST‐expressing (I) neurons during histamine stimuli. The conventions are the same as in Figure 2D‐E. Friedman test followed by simple effects analysis. n = 4 mice in both groups. (J–L) Ca^2+^ signals recorded from ACC glutamatergic J), PV‐expressing K), and SST‐expressing L) neurons during chloroquine stimuli. The conventions are the same as in (G–I). n = 4 mice in both groups. For glutamatergic neurons and SST‐expressing neurons, Friedman test followed by simple effects analysis; for PV‐expressing neurons, repeated ANOVA test. ***P < 0.001, **P < 0.01, *P < 0.05, NS: not significant.

We then examined the effects of acute itch stimuli on cingulate postsynaptic neuron subtypes of MD‐ACC projection neurons. First, FOS immunostaining showed that both histamine and CQ treatment increased FOS expression in EYFP‐labeled cingulate neurons in PV‐Cre mice, instead of CaMKIIα‐Cre and SST‐Cre mice (**Figure** **9**A–D). To validate the morphological results, we further combined the anterograde transsynaptic tracing strategy described above and the calcium imaging technique to monitor the calcium activity of these neurons in acute itch states, with rAAV‐EF1a‐Con/Fon‐GCaMP6s‐WPRE infused into the ACC (Figure 9E–F). The recordings in CaMKIIα‐Cre (Figure 9G,J) and SST‐Cre (Figure 9I,L) mice did not reveal any changes in GCaMP6s fluorescent signals before or after scratching induced by either histamine or CQ treatment. However, an increase in fluorescence signal was observed during the post‐scratching period in PV‐Cre mice after stimulation with histamine and CQ (Figure 9H,K). Thus, our observations from these diverse experimental techniques collectively suggest that only postsynaptic PV neurons in the MD‐ACC pathway are recruited upon acute itch stimulation.

## 3 Discussion

### 3.1 Neural Dynamics of Glutamatergic and GABAergic Neurons in the ACC during Acute Itch Processing

**Figure 10:**
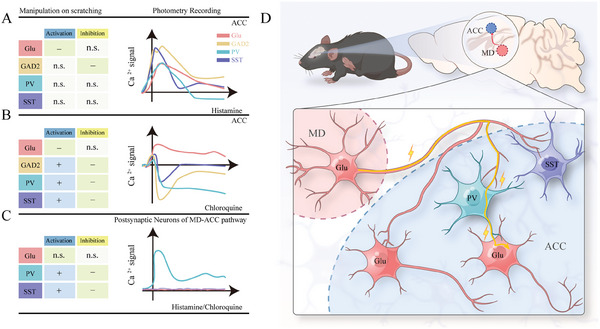
Summary of functional roles and neural dynamics of ACC neuron subpopulations during itch processing. A–B) Functional roles and neural dynamics of anterior cingulate cortex (ACC) glutamatergic and GABAergic neurons during histaminergic (A) and non‐histaminergic itch (B) processing. C) Functional roles and neural dynamics of postsynaptic glutamatergic and GABAergic neurons of the mediodorsal thalamic nucleus (MD)‐ACC pathway during histaminergic and non‐histaminergic itch processing. D) Model depicting the microcircuitry basis underlying the facilitatory role of the MD‐ACC pathway in acute itch processing. MD neurons project to glutamatergic, parvalbumin‐expressing, and somatostatin‐expressing neurons in the ACC. Under the condition of both histaminergic and non‐histaminergic itch, the MD‐ACC^PV^ pathway is exclusively recruited and mediates acute itch‐related scratching possibly by feedforward inhibition of ACC pyramidal neurons that did not receive inputs from the MD.

Similar to the pattern observed in other studies,^[^ ^10^, ^41^ ^]^ our fiber photometry data indicated that the activity changes in both anterior cingulate glutamatergic and GABAergic neurons occurred before the beginning of scratching and were maintained for a period afterwards. While the initial activity is proposed to reflect a sensory component of itching, prolonged calcium change is thought to result from both itching and scratching‐evoked mechanical sensations.^[^ ^10^, ^41^ ^]^ Activation of ACC glutamatergic neurons was observed under both types of acute itch stimuli, whereas the activity of GABAergic neurons differed with the time phase and itch modality (**Figure** **10**A–B). In CQ‐induced itching, a transient increase in Ca^2+^ signals was observed during the pre‐scratching period, followed by a sustained decrease in activity after scratching. Further recordings indicated that the SST‐INs were activated only during the pre‐scratching phase, whereas the PV‐INs were deactivated only during the post‐scratching phase. Thus, it is possible that SST‐INs and PV‐INs mediate the early and late phases of biphasic responses in GABAergic neurons, respectively. However, during histamine stimuli, GABAergic neurons show increased activity instead of the aforementioned biphasic bidirectional changes. Further analysis indicated that the dynamics of SST‐INs mimicked those of GABAergic neurons, whereas no significant change in calcium levels was observed in PV‐INs. Thus, SST‐INs may mediate calcium level changes in GABAergic neurons during the histaminergic itch state.

Accumulating preclinical evidence has shown that histamine‐dependent and‐independent itch modalities are conveyed through parallel peripheral pathways and separate neuropeptide‐encoding mechanisms within the SDH.^[^ ^42^ ^]^ Imaging studies have suggested the existence of overlapping but distinct brain networks that process these two types of itch.^[^ ^13^ ^]^ Although the ACC is activated under both itch conditions,^[^ ^13^ ^]^ emerging evidence suggests notable discrepancies in cellular encoding mechanisms between them, since both the AM‐ACC and ACC‐DMS pathways selectively modulate histaminergic itch instead of non‐histaminergic itch.^[^ ^19^, ^20^ ^]^ In our study, we found that the dynamics and modulatory roles of ACC neurons, especially GABAergic INs and their subpopulations, are distinct in these two types of itch. Thus, parallel GABAergic circuits may exist downstream of the pruritic inputs that modulate itch‐evoked scratching. Further application of state‐of‐the‐art techniques, including miniature two‐photon microscopy and dual patch clamp recordings, would provide a deeper understanding of itch‐related ACC microcircuitry mechanisms.

### 3.2 Functional Role of Glutamatergic and GABAergic Neurons in the ACC during Acute Itch Processing

Preclinical studies have shown that global inhibition of the ACC via pharmacological or chemogenetic techniques suppresses histamine‐induced scratching.^[^ ^17^, ^18^ ^]^ Corroborating these findings, our initial behavioral test showed that chemical ablation of the ACC, involving both glutamatergic and GABAergic neurons, inhibited both histamine‐ and CQ‐induced itch, suggesting that the ACC is a cortical hub for itch processing. Considering the lack of cell‐type specificity of these approaches, our subsequent experiments explored the diverse roles of ACC neuron subtypes in itch processing in a manner not easily predicted by the global manipulation approaches described herein and previously. We observed that specific inhibition of ACC GABAergic neurons suppressed acute itch‐related scratching, whereas inhibition of glutamatergic neurons did not. Thus, GABAergic neurons may mediate the facilitatory effects of the ACC on itch‐induced scratching.

In this study, we observed that ACC glutamatergic and GABAergic neurons exhibited opposite modulatory effects on itch processing because the activation of excitatory neurons or inactivation of GABAergic neurons led to decreased acute itch‐induced scratching (Figure 10A–B). Previous studies demonstrated that the activation of glutamatergic excitatory ACC neurons amplifies pain sensation, whereas GABAergic INs exert pain‐inhibitory effects mediated by PV‐INs instead of SST‐INs.^[^ ^23^, ^24^, ^43^, ^44^ ^]^ Thus, itch and pain appear to be intricately entangled. Noxious counterirritants reduce itching sensation, and suppression of pain may potentiate itch.^[^ ^45^ ^]^ Despite sharing similar neuroanatomical substrates for sensory transmission at the peripheral, spinal, and supraspinal sites, itch and pain are distinctly discriminated and processed at the cellular level. The circuitry underlying the inverse relationship between itch and pain has been implicated at the spinal level^[^ ^46^ ^]^ and in the descending control system.^[^ ^9^, ^10^, ^47^, ^48^ ^]^ Recent advances have raised the possibility that cortical mechanisms are also involved in this interaction. Spinal projecting neurons in the primary somatosensory cortex (S1) facilitate neuropathic pain processing within the SDH via postsynaptic excitatory INs. However, the same neuronal population exerts tonic inhibitory effects on itch transmission, which are mediated by postsynaptic GABAergic INs.^[^ ^49^, ^50^ ^]^ In our study, we identified another cortical area in which PYN activation affected itch and pain responses in an opposing manner. The ACC is assumed to regulate spinal sensory excitatory transmission via direct ACC‐SDH projections^[^ ^51^ ^]^ or through the descending PAG‐Rostral Ventromedial Medulla (RVM)‐SDH pathway.^[^ ^22^, ^52^ ^]^ Thus, different postsynaptic neuronal types within the SDH or PAG may mediate the opposing effects of ACC glutamatergic neurons on pain and itching.

While most of these behavioral results are internally consistent, we observed some asymmetric effects of cell type‐specific excitation and inhibition. First, the unidirectional effect of ACC neurons on acute itch stimuli was frequently encountered, which was exemplified in PYNs in both itch modalities and in GABAergic INs in the non‐histaminergic itch state, and these data were confirmed by chemogenetics and optogenetics in a complementary manner. Second, the differential roles of GABAergic neurons and their subtypes in histaminergic and non‐histaminergic itch further add to the complexity of cingulate itch modulation. Third, histaminergic itch‐induced scratching was suppressed by the inactivation of GABAergic neurons, but these effects could not be replicated by the optogenetic inactivation of PV^+^ and SST^+^ neurons. This is not entirely surprising, as PV‐ and SST‐INs represent ≈70% of the cortical GABAergic INs.^[^ ^26^, ^29^ ^]^ It is possible that vasoactive intestinal peptide‐expressing INs, another GABAergic subset occupying a pivotal node in cortical IN networks responsible for somatosensory modulation,^[^ ^53^, ^54^ ^]^ are also involved in histaminergic itch regulation. As with the activity changes illustrated above, ACC neurons also exhibited functional complexity during acute itch processing based on the dichotomy of glutamatergic and GABAergic subtypes (Figure 10A–B).

### 3.3 Functional Dissection of the MD‐ACC Pathway in the Modulation of Acute Itch

The difficulty in illustrating the above data concerning the dynamics and modulatory role of ACC neurons prompted us to reflect on the shortcomings of the methodology of current neuron‐type classification based on these cell markers and resorted to circuitry‐based ideology to decipher the microcircuitry mechanisms underlying cingulate itch modulation. In pain research, hyperactivity of ACC PYNs has been shown to drive the aversion associated with chronic pain.^[^ ^55^, ^56^ ^]^ However, a recent study by Meda et al.^[^ ^25^ ^]^ reported that the MD‐ACC pathway exacerbated pain‐related aversion, whereas amygdaloid inputs to the ACC had the opposite effect, suggesting that chronic pain‐associated aversion reflects activity changes in specific pathways rather than generalized ACC hyperactivity. Although global activation of ACC glutamatergic neurons promoted acute itch‐related scratching, we found no evidence for the role of postsynaptic PYNs in MD‐ACC projections in itch modulation. In addition, Lu et al. reported that photoactivation of ACC‐striatum projections increases histaminergic itch‐induced scratching.^[^ ^19^ ^]^ Based on these findings, the group of glutamatergic neurons demonstrated functional heterogeneity in itch processing, which could be clarified based on connectivity features. With the aid of combined cell type‐ and projection‐specific manipulations, we clarified the functional role of cingulate postsynaptic neuron subtypes of the MD‐ACC pathway, which were conserved across two different models of acute itch (Figure 10C).

Feedforward inhibition mediated by cortical inhibitory INs^[^ ^57^, ^58^ ^]^ is a key feature of thalamocortical interactions and is thought to control the temporal precision of cortical responses to sensory stimuli.^[^ ^59^ ^]^ For the MD‐ACC pathway, prior electrophysiological data have shown that MD inputs elicited both excitation and feedforward inhibition in ACC PYNs, and this feedforward inhibition was mediated by local PV‐INs rather than SST‐INs.^[^ ^59^ ^]^ Interestingly, our morphological data presented here and before^[^ ^39^ ^]^ suggest that MD preferentially innervates postsynaptic PV neurons within this thalamocotical circuit. In a recent study, we further provided morphological and electrophysiological evidence showing that hyperactivated MD preferentially recruited postsynaptic PV+ neurons (not their glutamatergic and SST‐expressing counterparts) under the condition of post‐traumatic stress disorder (PTSD), which played a critical role in mediating abnormal fear extinction.^[^ ^39^ ^]^ In this study, we observed that postsynaptic PV‐ and SST‐INS, instead of their glutamatergic counterparts, of this pathway promoted acute itch‐induced scratching. However, only PV‐INs were activated in acute itching (Figure 10C). On the basis of these evidence, we speculated that the postsynaptic PV+ neurons might predominate cortical processing and regulatory mechanisms of negative external stimuli by the MD‐ACC pathway. In addition, it is reasonable to propose that the recruitment of postsynaptic PV‐INs mediates the itch‐facilitatory role of the MD‐ACC pathway, possibly via feedforward inhibition of ACC PYNs that do not receive inputs from the MD (Figure 10D). It is, therefore, of interest for future studies to investigate this specific PYN subtype and the downstream circuitry mechanisms through which PYN inhibition suppresses the itch sensation.

### 3.4 Limitations and Summary

The MD‐prefrontal cortex (PFC) circuit supports higher‐order cognitive functions.^[^ ^60^ ^]^ However, the role of MD inputs to the ACC, a cortical area sharing anatomical and functional similarity with the PFC,^[^ ^61^ ^]^ in cognitive control has not been touched. With the development of neurocircuitry decoding techniques, we are delighted to see that the role of this pathway in driving aversion associated with chronic pain and fear extinction impairment in post‐traumatic stress disorder has been unveiled.^[^ ^25^, ^39^ ^]^ Given above, this pathway might convey information associated with external negative stimuli preferentially concerning the emotional valence. In this study, we proved that the MD‐ACC pathway contributed to cortical itch processing in the sensory aspect, and its involvement in the emotional dimension of itch remains an important topic. In addition, chronic itching induces the potentiation of synaptic transmission in the ACC.^[^ ^62^ ^]^ It remains unclear whether chronic itch remodels the MD‐ACC pathway to modify itch sensitivity.

This study revealed a previously unknown causal link between the activity of distinct ACC cell types and itch‐related behavioral outputs and delineated a thalamocortical circuit originating from the MD that was critically involved in histaminergic and non‐histaminergic itch processing. These data highlight the general role of the ACC in coordinating pruritic behaviors and provide clues for developing therapeutic strategies for itch‐related diseases. Recent studies have identified the ACC as a novel promising target for the management of both sensory and affective aspects of chronic pain in the domain of neuromodulation.^[^ ^63^, ^64^ ^]^ It will be exciting for future studies to target the ACC, even specific cell types or inputs, in the treatment of chronic itch.

## 4 Experimental Section

### Experimental Design and Virus Injection

The mice were anesthetized with isoflurane (4% for induction and 1.5% for maintenance), and their heads were fixed in a stereotaxic injection frame (RWD Life Science Inc., China). Injections into the ACC or MD were performed using a microinjection needle with a 10 µL microsyringe (Shanghai Gaoge Industry & Trade Co., LTD, China) to deliver the virus or the drug at a rate of 30 nL min^−1^ using a microsyringe pump (KD Scientific Inc., USA). Following injection, the needle was held at the site for another 10 min to allow diffusion. The stereotaxic coordinates for the ACC injection were as follows: anterior‐posterior (AP), 0.80; medial lateral (ML), 0.30; and dorsal‐ventral (DV), 1.75 mm, while those for MD were AP, −1.1; ML, 0.50; DV, 3.05 mm.

For pharmacological lesion of the ACC, a 0.5 uL volume of a 0.1 M solution of quinolinic acid (Cat#: HY‐100807, MedChemExpress, USA) in saline was administered into each side of the ACC. The control mice were injected with saline in the same manner. Behavioral assays were performed seven days later.

To monitor the neuronal activity of ACC glutamatergic neurons under acute itch stimuli, C57BL/6J mice were injected with 200 nL of rAAV2/9‐CaMKIIa‐GCaMP6s‐WPRE (Cat#: PT‐0110, titer: 2.66 × 10^12^ vg mL^−1^, BrainVTA) or rAAV2/9‐CaMKIIa‐EYFP‐WPRE (titer: 2.36 × 10^12^ vg mL^−1^; Cat#: PT‐0107, BrainVTA) as controls in the right ACC of C57BI/6J mice. To record the activity of GABAergic, PV, and SST^+^ neurons in the ACC, 200 nL of rAAV2/9‐hSyn‐DIO‐GCaMP6s‐WPRE (titer: 5.40 × 10^12^ vg mL^−1^; Cat#: PT‐0091, BrainVTA) or rAAV2/9‐hSyn‐DIO‐EGFP‐WPRE (titer: 2.31 × 10^12^ vg mL^−1^; Cat#: PT‐1103, BrainVTA) as a control was injected into the right ACC of GAD2‐Cre, PV‐Cre, and SST‐Cre mice, respectively. Fiber photometry was performed three weeks later.

To monitor the neuronal activity of ACC glutamatergic neurons under the condition of local GABAergic subpopulation activation, PV‐Cre and SST‐Cre mice were injected with a mixture of rAAV‐CaMKIIa‐GCaMP6s‐WPRE (50 nL) and rAAV‐hSyn‐DIO‐ChrimsonR‐mCherry (titer: 2.50 × 10^12^ vg mL^−1^, Cat#: BC‐0220, Braincase) or rAAV‐EF1α‐DIO‐eNpHR3.0‐mCherry‐WPRE (titer: 2.55 × 10^12^ vg mL^−1^; Cat#: PT‐0007, BrainVTA) (150 nL) into the right ACC. Two weeks later, an optic fiber was implanted into the ACC for simultaneous photostimulation and recording.

For optogenetic manipulation of ACC glutamatergic neurons, rAAV2/9‐CaMKIIa‐hChR2(H134R)‐mCherry‐WPRE (titer: 5.72 × 10^12^ vg mL^−1^; Cat#: PT‐0297, BrainVTA) or rAAV2/9‐CaMKIIa‐eNpHR3.0‐mCherry‐WPRE (titer: 5.16 × 10^12^ vg mL^−1^; Cat#: PT‐0009, BrainVTA) was injected bilaterally into the ACC at a volume of 200 nL of C57BL/6J mice. rAAV2/9‐CaMKIIa‐EYFP‐WPRE (titer: 2.31 × 10^12^ vg mL^−1^; Cat#: PT‐1103, BrainVTA) was used as a control. For optogenetic manipulation of GABAergic, PV‐expressing, and SST‐expressing neurons in the ACC, rAAV2/9‐EF1α‐DIO‐hChR2(H134R)‐EYFP‐WPRE (titer: 2.73 × 1012 vg mL^−1^; Cat#: PT‐0001, BrainVTA) or rAAV2/9‐EF1α‐DIO‐eNpHR3.0‐mCherry‐WPRE was injected bilaterally into the ACC at a volume of 200 nL of GAD2‐Cre, PV‐Cre, and SST‐Cre mice, respectively. rAAV2/9‐hSyn‐DIO‐EGFP‐WPRE (titer: 2.31 × 10^12^ vg mL^−1^; Cat#: PT‐1103, BrainVTA) was used as the control. Optic fiber was implanted into bilateral ACC two weeks after virus injection and behavior essays were performed one week later.

For evaluation of the effect of optical manipulation of the MD‐ACC pathway on acute itch‐induced scratching behavior, 160 nL rAAV2/9‐hSyn‐hChR2(H134R)‐mCherry‐WPRE (titer: 5.08 × 10^12^ vg mL^−1^; Cat#: PT‐0150, BrainVTA) or 160 nL rAAV2/9‐hSyn‐eNpHR3.0‐mCherry‐WPRE (titer: 6.00 × 10^12^ vg mL^−1^; Cat#: PT‐0011, BrainVTA) was injected into the bilateral MD (80 nL on each side), with the same amount of rAAV2/9‐hSyn‐EYFP‐WPRE (titer: 5.26 × 10^12^ vg mL^−1^; Cat#: PT‐0102, BrainVTA) injected as a control. Optic fiber was implanted into bilateral ACC two weeks after virus injection and behavior essays were performed one week later.

For chemogenetic manipulation of ACC glutamatergic neurons, rAAV2/9‐CaMKIIa‐hM3D(Gq)‐mCherry‐WPRE (titer: 5.29 × 10^12^ vg mL^−1^; Cat#: PT‐0049, BrainVTA) or rAAV2/9‐CaMKIIa‐hM4D(Gi)‐EFGP‐WPRE (titer: 4.90 × 10^12^ vg mL^−1^; Cat#: PT‐0524, BrainVTA) was injected bilaterally into the ACC at a volume of 200 nL of C57BL/6J mice. rAAV2/9‐CaMKIIa‐EYFP‐WPRE was used as a control. For chemogenetic manipulation of ACC GABAergic neurons, a 200 nL mixture of rAAV2/9‐EF1α‐DIO‐hM3D(Gq)‐mCherry‐WPRE (titer: 2.36 × 10^12^ vg mL^−1^; Cat#: PT‐0042, BrainVTA) or rAAV2/9‐EF1α‐DIO‐hM4D(Gi)‐mCherry‐WPRE (titer: 2.40 × 10^12^ vg mL^−1^; Cat#: PT‐0043, BrainVTA) was injected bilaterally into the ACC of GAD2‐Cre mice. rAAV2/9‐hSyn‐DIO‐EGFP‐WPRE was used as a control. For chemogenetic manipulation of MD neurons, a 80 nL mixture of rAAV2/9‐hSyn‐hM3D(Gq)‐EGFP‐WPRE (titer: 5.45 × 10^12^ vg mL^−1^; Cat#: PT‐0152, BrainVTA) or rAAV2/9‐hSyn‐hM4D(Gi)‐EGFP‐WPRE (titer: 2.40 × 10^12^ vg mL^−1^; Cat#: PT‐0153, BrainVTA) was injected bilaterally into the MD of C57BL/6J mice. rAAV2/9‐hSyn‐EYFP‐WPRE (titer: 5.26 × 10^12^ vg mL^−1^; Cat#: PT‐0102, BrainVTA) was used as a control. Behavioral assays were performed three weeks after virus injection.

For whole‐brain mapping of long‐range direct inputs to glutamatergic, PV‐expressing, and SST‐expressing neurons in the ACC, 200 nL AAV helper viruses were injected into the right ACC in CaMKIIα‐Cre, PV‐Cre or SST‐Cre mice, respectively, mixed with rAAV2/9‐EF1α‐DIO‐EGFP‐T2A‐TVA‐WPRE (titer: 3.40 × 10^12^ vg mL^−1^; Cat#: PT‐0023, BrainVTA) and rAAV2/9‐EF1α‐DIO‐EGFP‐T2A‐TVA‐WPRE (titer: 3.39 × 10^12^ vg mL^−1^; Cat#: PT‐0062, BrainVTA) at a ratio of 1:2. One month later, 200 nL of RV‐ENVA‐ΔG‐dsRed (titer: 2.00 × 10^8^ IFU mL^−1^; Cat#: R01002, BrainVTA) was injected into the same site. Only with the help of TVA and RG can RV‐EGFP spread retrogradely to presynaptic neurons. One week after the RV injection, the mice were perfused for morphological observations. To quantitatively compare the input amounts in different subregions, the input neurons in each upstream region were counted and normalized to the total number of starter cells.

Transsynaptic anterograde tracing was used to specifically label the cingulate postsynaptic neuron subtypes of MD‐ACC projection neurons. A mixture of 80 nL rAAV2/1‐hSyn‐FLP‐WPRE (titer: 1.02E+13 vg mL^−1^; Cat#: PT‐0341, BrainVTA) and 10 nL CTB555 (for marking the injection site) were injected into the right MD and 180 nL rAAV2/9‐hSyn‐Con/Fon‐EYFP‐WPRE‐pA (titer: 2.24 × 10^12^ vg mL^−1^; Cat#: PT‐0169, BrainVTA) into the right ACC in CaMKIIα‐Cre, PV‐Cre mice, and SST‐IRES‐Cre mice. For optogenetic modulation of these specific postsynaptic neuron subtypes, 160 nL rAAV2/1‐hSyn‐FLP‐WPRE was injected into bilateral MD (80 nL in each side) and 400 nL rAAV2/9‐hSyn‐Con/Fon‐eNpHR3.0‐EYFP‐WPRE (titer: 5.30 × 10^12^ vg mL^−1^; Cat#: PT‐1154, BrainVTA), rAAV2/9‐hSyn‐Con/Fon‐hChR2(H134R)‐EYFP‐WPRE (titer: 1.30E+13 vg mL^−1^; Cat#: PT‐0065, BrainVTA), or rAAV2/9‐hSyn‐Con/Fon‐EYFP‐WPRE into the bilateral ACC (200 nL in each side) in these transgenic mice. For monitoring the calcium activity of these neurons, 80 nL rAAV2/1‐hSyn‐FLP‐WPRE was injected into the right MD and 200 nL rAAV2/9‐EF1a‐Con/Fon‐GCaMP6s‐WPRE (titer: 5.75 × 10^12^ vg mL^−1^; Cat#: PT‐1553, BrainVTA) or rAAV2/9‐hSyn‐Con/Fon‐EYFP‐WPRE into the right ACC in CaMKIIα‐Cre, PV‐Cre mice, and SST‐Cre mice.

### Itch Behavioral Test

For acute itch testing, the mice were shaved on the back of the neck and individually handled daily for five days before the behavioral tests. During the tests, the mice were briefly removed from the chamber and intradermally injected with the pruritic compounds, histamine (100 µg/10 µL, Cat#: H7125, Millipore Sigma, USA), or CQ (100 µg/10 µL, Cat#: C6628, Millipore Sigma) into the nape. The animals were videotaped, and scratching behavior was manually counted in a blinded manner.

For the establishment of chronic itch, the contacted dermatitis model was adopted. The mice were shaved on the back and topically applied by painting 0.2 mL of 1% diphenylcyclopropenone (DCP, Cat#: 177 377, Millipore Sigma, USA) dissolved in acetone under isoflurane anesthesia. Seven days after the first painting, 0.2 mL of 0.5% DCP was painted again on the same area of skin for three days. Three days later, scratching behavior was recorded as described before.

### Chemogenetic Manipulations

Three weeks after the DREADDs injection, the mice were intraperitoneally administered a 0.25 mg mL^−1^ solution of CNO (4 µL g^−1^; Cat#:4936, Tocris) in saline. The mice were immediately transferred to recording cages, where recordings were initiated after 1 h. For evaluating the effect of chemogenetic manipulation of ACC glutamatergic and GABAergic neurons as well as MD neurons on acute itch‐induced scratching, the mice received histamine or CQ injection before recording. In another batch of mice for evaluating the effect of chemogenetic manipulation on spontaneous scratching, the mice behavior was directly recorded for 10 min without any treatment. After behavioral tests, the mice were perfused and those with viral expression restricted to the ACC were chosen for statistical analysis. FOS immunostaining was performed to validate the efficacy of the chemogenetics.

### Optogenetic Manipulations

Three weeks after the virus injection, optical fibers (optical density: 230 µm; numerical aperture: 0.37; Newdoon, China) were implanted in bilateral ACC. The stereotaxic coordinates for implantation were AP 0.80, ML 0.75, and DV 1.25 mm, with a 15° tilt angle of the optical fiber to the vertical line. Four weeks after the virus injection, an external optical fiber was used to connect a 473 nm or 589 nm laser power source (Newdoon) to the optical fiber implanted in the animal. The laser pulses were controlled using an arbitrary waveform generator (AFG3000C, Tektronix, Inc. USA). For optogenetic inhibition, yellow light (589 nm) was delivered in a continuous pattern at 3‐min intervals (with a 3‐min light‐on period followed by a 3‐min light‐off period). The final output power of the light ranged from 6 to 9 mW depending on the light transmission efficacy of the optical fiber used. These pulses were repeated for 30 min after the histamine or CQ treatment. However, the photoactivation parameters differed depending on the experimental design. For the behavioral assays, we adopted a phasic excitation protocol using blue light (473 nm, 40 ms pulse width, 20 Hz, 5–8 mW). For simultaneous calcium recordings, yellow light (589 nm, 40 ms pulse width, 20 Hz, 5–8 mW) was used for photoactivation, and yellow light (589 nm) was delivered in a continuous pattern similar to that of the optogenetic inhibition described above. After the tests, the mice were perfused and those with viral expression and optical fiber tips restricted to the ACC were chosen for statistical analysis. FOS immunostaining was performed to validate the efficacy of the optogenetics.

### Fiber Photometry

The mice were allowed to recover for two weeks after the injection of the AAV expressing GCaMP6s or EYFP. Next, each mouse was implanted with an optical fiber (230 µm OD, 0.5 NA; Newdoon) in the right ACC with the same coordinates as those of the virus injection. A skull‐penetrating screw and dental acrylic were used to fix the ceramic ferrule. The mice were handled one week before fiber photometry recording. Calcium signals were recorded using a commercial fiber photometry system (ThinkerTech Nanjing Bioscience Inc., China) as previously described.^[^ ^65^ ^]^ To record the fluorescence signals, a 473 nm laser beam (OBIS 48LS; Coherent) was reflected off a dichroic mirror (MD498, Thorlabs Inc., USA) that was focused using a 10 × 0.3 NA objective lens (Olympus, Japan) coupled to an optical commutator (Doris Lenses, Canada). An optical fiber (230 µm OD, 0.5 NA) guided the light between the commutator and the implanted optical fiber. The laser power at the tip of the optical fiber was adjusted to 0.01–0.02 mW to reduce laser bleaching. The fluorescence was bandpass filtered (MF525‐39, Thorlabs Inc., USA), and an amplifier was used to convert the CMOS (DCC3240M, Thorlabs) current output to a voltage signal, which was further filtered through a low‐pass filter (40 Hz cutoff; ThinkerTech). Analog voltage signals were digitalized at 50 Hz and recorded using a multichannel fiber photometry recording system (ThinkerTech). The fluorescence signals were recorded continuously during scratching. After the behavioral tests, the mice were perfused and those with viral expression and optical fiber tips restricted to the ACC were included for further analysis.

For data analysis, the fluorescence change (ΔF/F) was calculated as (F‐F_0_)/F_0_, where F refers to the fluorescence values at each time point and F_0_ denotes the median of the fluorescence values in the baseline period. The ΔF/F values of the animals in each group were averaged. To precisely analyze the changes in fluorescence values across the scratching train, we defined the baseline period (−5 to −3 s relative to the behavioral onset), pre‐scratching period (−2 to 0 s relative to onset), and post‐scratching period (0–2 s after the onset of scratching behaviors). To quantify the change in the level of the calcium signal induced by the itch sensation, the area under the curve of ΔF/F in each time window was calculated.

To test whether the fluorescence changes within specific neuronal populations in the ACC were related to locomotor bouts not associated with scratching, we further analyzed the changes in fluorescence values across the trains of digging, grooming, walking, and standing under the condition of acute itch induced by CQ. In this analysis, we defined the baseline period (−5 to −3 s relative to the behavioral onset) and post‐locomotion period (0–2 s after the onset of locomotion).

### Immunofluorescent Staining

The mice were anesthetized with an overdose of 2% pentobarbital sodium and perfused transcardially with saline, followed by 4% paraformaldehyde (PFA, Millipore Sigma) in phosphate‐buffered saline (PBS). Their brains were dissected and post‐fixed overnight at 4 °C in 4% PFA, followed by cryoprotection in 30% sucrose in PBS at 4 °C. Free‐floating sections (30 µm) prepared with a cryostat (Leica CM 1950, Leica Microsystems Inc., USA) were used for immunohistochemical staining. Tissue sections were blocked for 30 min at room temperature (22‐24 °C) in PBST (0.3% Triton X‐100) containing 5% normal donkey serum. This was followed by incubation with primary antibodies at 4 °C overnight, and with secondary antibodies (with DAPI, 1:1000, Cat#: D9564, Millipore Sigma) at room temperature for 4 h. Finally, the sections were air‐dried and cover‐slipped with a mixture of 50% (v/v) glycerol and 2.5% (w/v) triethylenediamine in 0.01 M PBS. Photomicrographs of the brain sections were captured using an Olympus VS200 microscope (10×), and confocal images of the ACC were captured using an Olympus fv3000 microscope (40×). Cells were counted manually in a blinded manner.

To examine FOS expression in ACC glutamatergic neurons, C57BL/6J mice were sacrificed 90 min after the intradermal injection of histamine or CQ, and double immunofluorescence of FOS and CaMKII was performed. The primary antibodies used for immunohistochemistry (IHC) were rabbit anti‐CaMKII (1:200, Cat#: ab5683, Abcam, USA) and mouse anti‐Fos B (1:500, Cat#: ab11959, Abcam). The secondary antibodies used were donkey anti‐rabbit IgG‐Alexa Fluor 488 (1:500, Cat#: ab150073, Abcam) and donkey anti‐mouse IgG‐Alexa Fluor 594 (1:500, Cat#: A21203, Invitrogen, USA). To examine FOS expression in ACC GABAergic neurons, GAD67‐GFP transgenic mice were euthanized 90 min after histamine or CQ stimulation. Since GAD67 was manifested by the expression of GFP, only FOS immunostaining was performed. The primary and secondary antibodies were mouse anti‐Fos B (1:500) and donkey anti‐mouse IgG‐Alexa 594 (1:500), respectively.

To confirm the presence of chemical lesions in the ACC, C57BL/6J mice were euthanized immediately after the behavioral tests. Double immunofluorescence staining for NeuN (a neuronal marker) and glial fibrillary acidic protein (GFAP; an astrocytic marker) was performed. The primary antibodies used were mouse anti‐NeuN (1:500, Cat#: ab104224, Abcam) and rabbit anti‐GFAP (1:200, Cat#: Z0334, Dako, Agilent Technologies, Inc., USA). The secondary antibodies were donkey anti‐rabbit IgG‐Alexa Fluor 488 (1:500) and donkey anti‐mouse IgG‐Alexa Fluor 594 (1:500).

To examine the efficacy of optogenetics and chemogenetics in different mouse lines, mice (C57BL/6J, GAD2‐Cre, PV‐Cre, and SST‐Cre mice) were euthanized immediately after the optogenetic and chemogenetic experiments, and FOS immunofluorescence staining was performed. The primary antibody used was mouse anti‐Fos B (1:500, Cat#: ab11959, Abcam), and the secondary antibodies were donkey anti‐mouse IgG‐Alexa Fluor 594 (1:500, Cat#: A21203, Invitrogen) or goat anti‐mouse IgG‐Alexa Fluor 488 (1:500, Cat#: KT‐00060, Invitrogen). FOS expression was observed in virus‐labeled neurons, as demonstrated by mCherry or EYFP.

To confirm the specificity of AAV‐GCaMP6s, mice (C57BL/6J, GAD2‐Cre, PV‐Cre, and SST‐Cre mice) were euthanized after fiber photometry. Because the AAV‐GCaMP6s contained a GFP tag, we performed only single immunostaining for the different neuronal markers. The primary antibodies used were rabbit anti‐CaMKII (1:200), rabbit anti‐PV (1:200, Cat#: ab11427, Abcam), or rabbit anti‐SST (1:200, Cat#: 20 067, ImmunoStar Inc., USA), and the secondary antibody was donkey anti‐mouse IgG‐Alexa Flour 594 (1:500).

### Quantification and Statistical Analysis

Data are expressed as the mean ± SEM. Data were analyzed using GraphPad Prism 8.0 software and SPSS 26.0 software (IBM, United States). The raw data and details of particular statistical analyses were provided in Tables S1‐S3 (Supporting Information). Briefly, the normality and the homogeneity of variance tests were performed with the Shapiro‐Wilk test and Levene's test, respectively. Data that met these two conditions were analyzed using a two‐tailed, unpaired, or paired t‐test, one‐factor analysis of variance (ANOVA) followed by Bonferroni correction for post hoc test, and repeated‐measures ANOVA followed by Simple effects analysis. Data sets that were not normally distributed were analyzed with a nonparametric test. A P‐value of less than 0.05 was considered statistically significant.

### Study Approval

All experimental procedures were approved by the Institutional Animal Care and Use Committee of the Fourth Military Medical University (FMMULL‐20220928) and conformed to the Guide for the Care and Use of Laboratory Animals published by the National Institutes of Health. All mice were maintained under a 12‐h light/dark cycle at 22–25 °C with ad libitum access to food and water under environmentally controlled conditions. All the animals used in this study were adult males with a pure C57BL/6J background. The C57BL/6J mice were purchased from the Experimental Animal Center of the Fourth Military Medical University. The CaMKIIα‐Cre (Stock No: 0 05359), PV‐IRES‐Cre (Stock No: 0 08069), SST‐IRES‐Cre (Stock No: 01 3044), and GAD2‐IRES‐Cre (Stock No: 01 0802)^[^ ^66^ ^]^ mice were obtained from the Jackson Laboratory. The GAD67‐GFP knock‐in mouse line was a gift from the Department of Morphological Brain Science, Kyoto University.^[^ ^67^ ^]^ All viral injections were administered to mice aged 2 months old, and all behavioral tests were carried out during the light phase. The experimenters were blinded to the genotypes and experimental conditions.

## Conflict of Interest

The authors declare no conflict of interest.

## Supporting information

Supporting Information

Supplemental Table 1

## Data Availability

The data that support the findings of this study are available in the supplementary material of this article.
