# Supplementary material for: Cell Type‐Specific Modulation of Acute Itch Processing in the Anterior Cingulate Cortex
Source: Adv Sci (Weinh). 2024 Sep 24;11(43):2403445. doi: 10.1002/advs.202403445 (PMC11578322; doi:10.1002/advs.202403445)
Supplement: Supplementary file 1 — Supporting Information [file ADVS-11-2403445-s002.docx]

Supporting Information

Cell type-specific modulation of acute itch processing in the anterior cingulate cortex

Jiaqi Li^1, 3†^, Yang Bai^2†^, Junye Ge^1†^, Yi Wen Zhang^1†^, Qiuying Zhao^1^, Dangchao Li^1^, Baolin Guo^1^, Shasha Gao^1^, Yuanyuan Zhu^1^, Guohong Cai^1^, Xiangdong Wan^1^, Jing Huang^1*^, Shengxi Wu^1*^


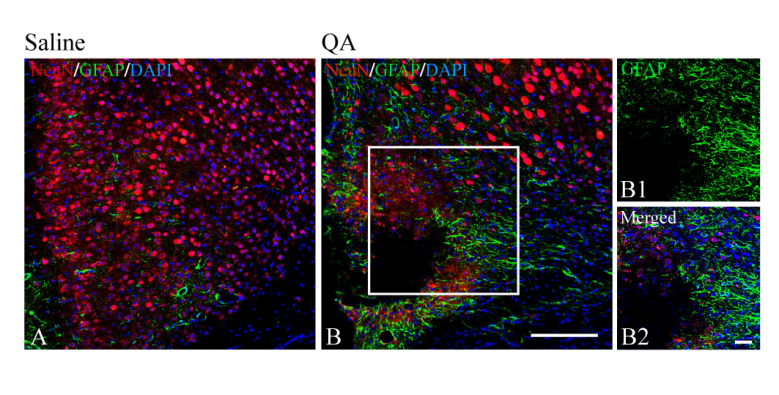


**Figure S1.** Local quinolinic acid injection results in neural loss and gliosis in the ACC. (A–B) Immunostaining of glial fibrillary acidic protein (GFAP, green) and NeuN (red) around the injection site in the ACC of mice injected with vehicle (A) or QA (B). The box in B is further magnified in B1 and B2. Scale bars: 200 μm (A and B) and 50 μm (B1 and B2). ACC, anterior cingulate cortex; QA, quinolinic acid.


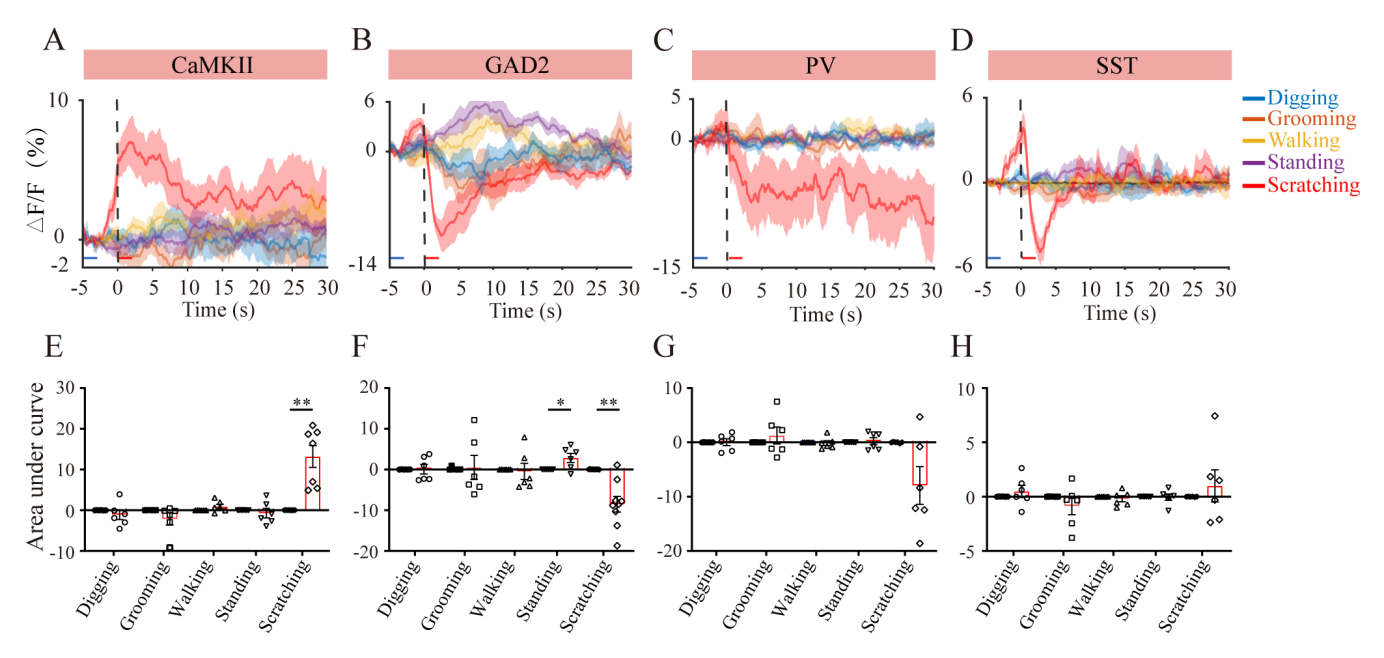


**Figure S2.** Activity of ACC neuronal subpopulations during locomotor bouts under the condition of chloroquine treatment. (A-D) Ca^2+^ signals recorded from ACC CaMKII-expressing (A), GAD2-expressing (B), PV-expressing (C), and SST-expressing (D) neurons during locomotor bouts in mice receiving AAV-GCaMP6s injection in the ACC. (E-H) Area under the curve showing changes in GCaMP6s fluorescence of ACC CaMKII-expressing (E), GAD2-expressing (F), PV-expressing (G), and SST-expressing (H) neurons in the baseline value and post-locomotion periods under the influence of CQ stimuli. Wilcoxon signed-rank test or paired t-test. N = 9 mice for analyzing scratching behaviors and 6 for analyzing digging, grooming, walking, and standing behaviors. **P < 0.01, *P < 0.05.

**
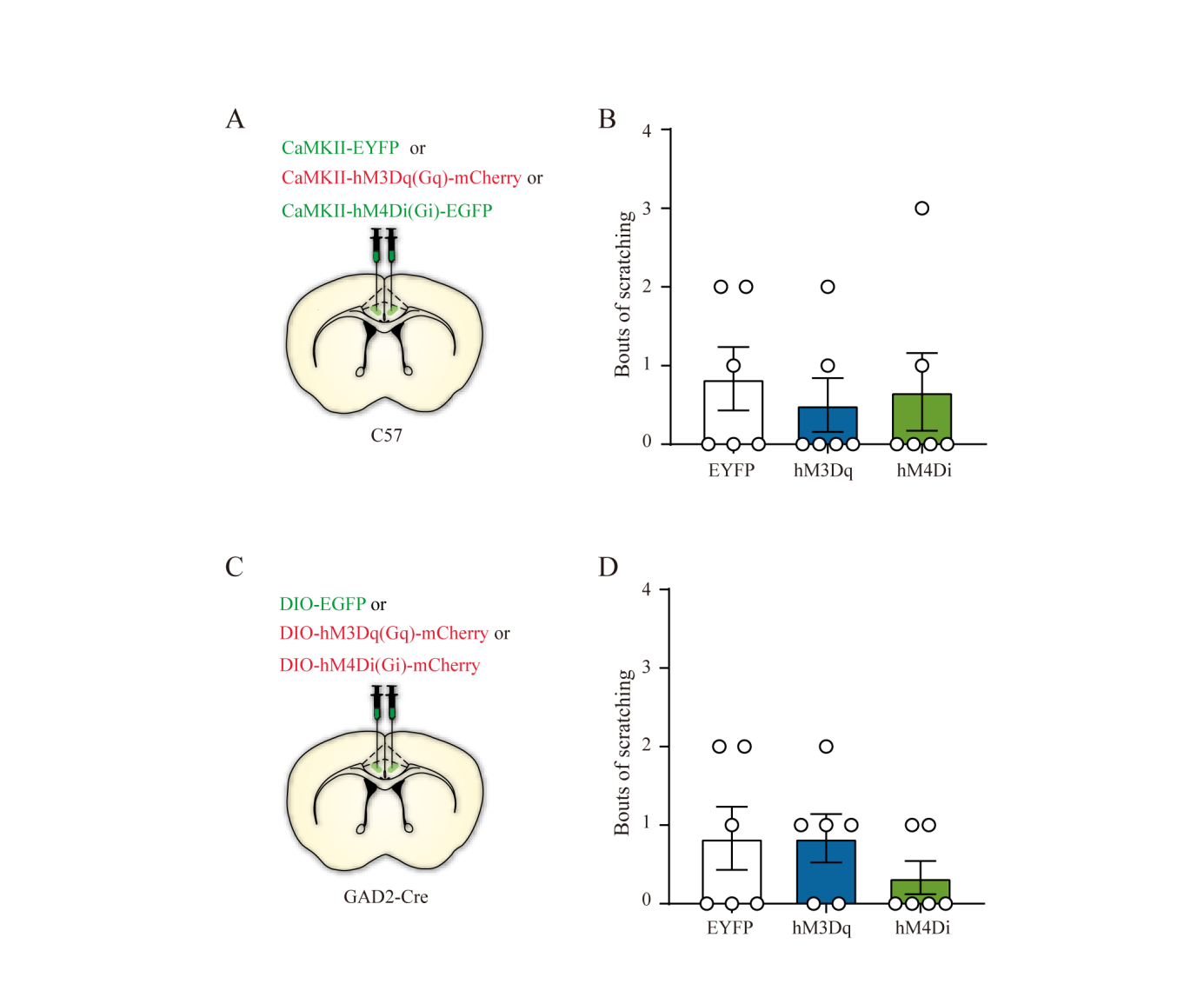
**

**Figure S3.** Effects of chemogenetic manipulation of ACC glutamatergic and GABAergic neurons on spontaneous scratching behavior. (A) Schematic illustration of virus injection for chemogenetic manipulation of ACC glutamatergic neurons. (B) Chemogenetic activation or inactivation of ACC glutamatergic neurons did not affect spontaneous scratching behavior in mice. N = 6 mice in each group. Kruskal-Wallis H test. (C) Schematic illustration of virus injection for chemogenetic manipulation of ACC GABAergic neurons. (D) Chemogenetic activation or inactivation of ACC GABAergic neurons did not affect spontaneous scratching behavior in mice. N = 6 mice in each group. Kruskal-Wallis H test.


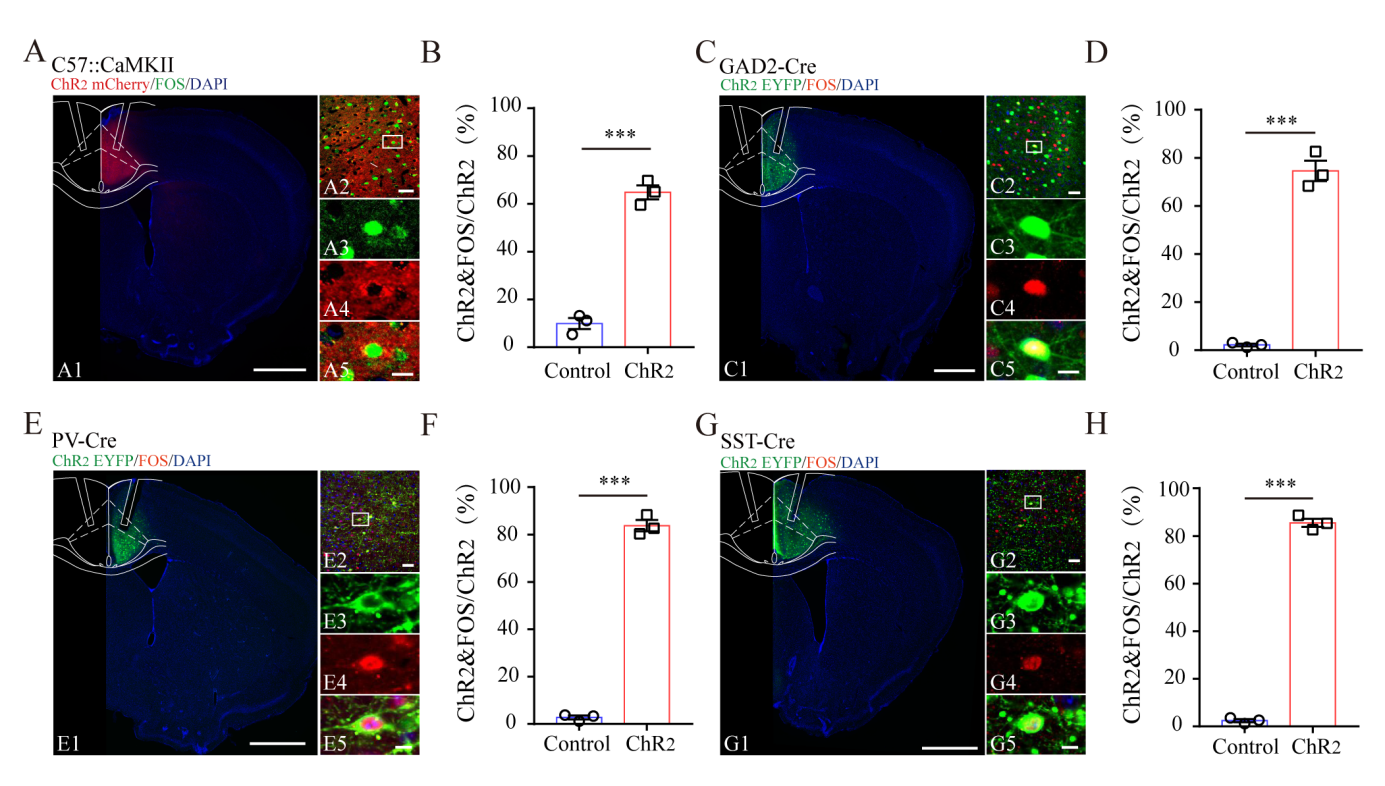


**Figure S4.** Verification of the efficacy of optogenetics in behavioral assays via FOS staining. (A) Histological verification of viral expression within the anterior cingulate cortex in a representative mouse injected with adeno-associated virus (AAV)-CaMKII-ChR2-mCherry (left) and FOS expression (green) in a representative mCherry-labeled neuron (red) in response to blue light illumination (right). The framed areas in A2 are magnified in A3–5. Scale bars: 1 mm (A1), 50 μm (A2), and 5 μm (A3–5). (B) Blue light illumination increases the expression of FOS in mCherry-labeled neurons in mice injected with AAV-CaMKII-ChR2-mCherry compared to control mice. n = 3 mice per group, 3 sections per animal. Unpaired *t*-test. (C–D) Blue light illumination increases the expression of FOS in GAD2-Cre mice injected with AAV-DIO-ChR2-mCherry compared to that in control mice. Unpaired *t*-test. (E–F) Blue light illumination increases the expression of FOS in PV-Cre mice injected with AAV-DIO-ChR2-mCherry compared to that in control mice. Unpaired *t*-test. (G–H) Blue light illumination increases the expression of FOS in SST-Cre mice injected with AAV-DIO-ChR2-mCherry compared to that in control mice. Unpaired *t*-test. ****P* < 0.001.


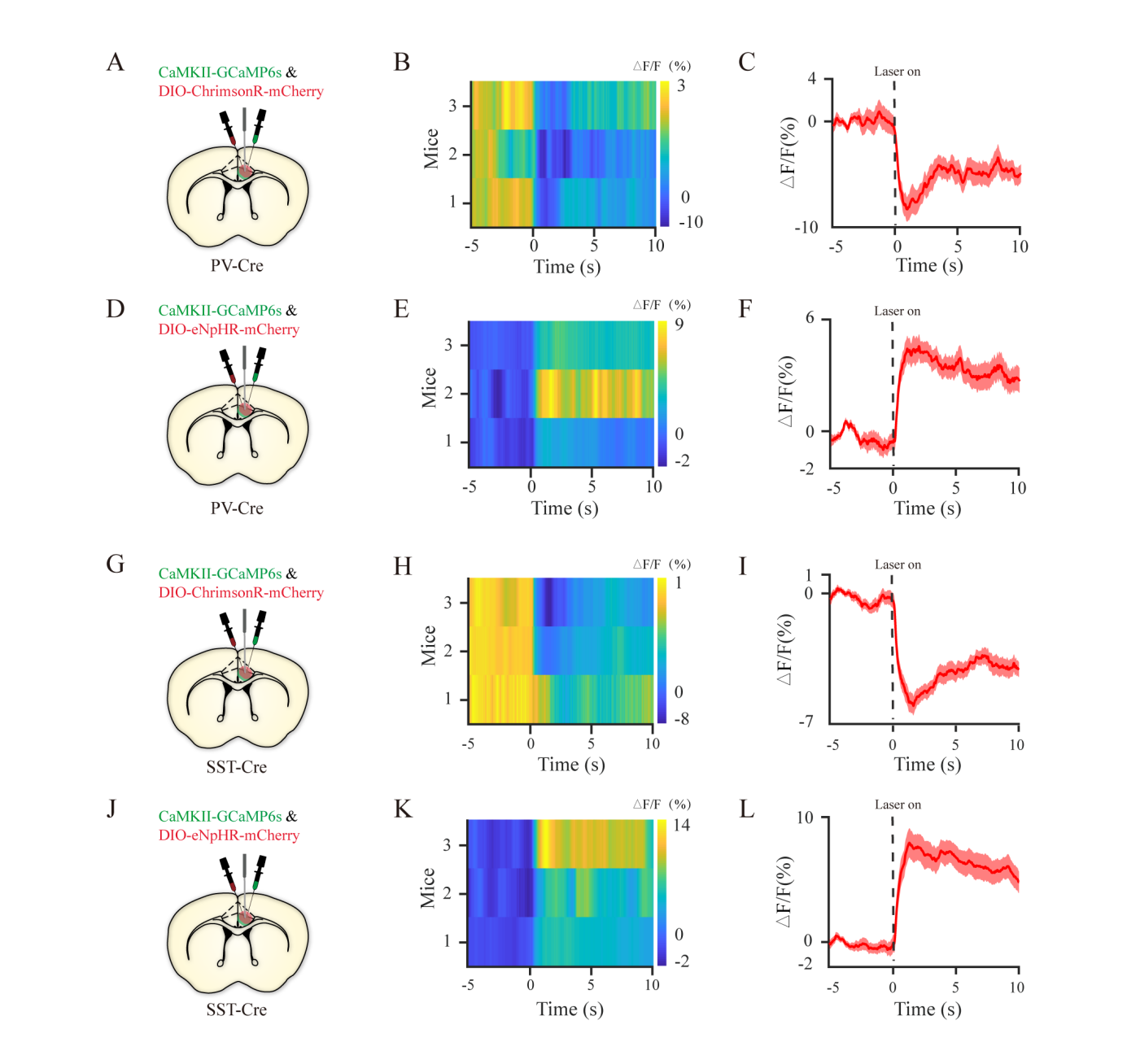


**Figure S5.** Effects of optogenetic manipulation of ACC PV- and SST-expressing neurons on the basal activity of glutamatergic neurons. (A) Schematic showing the experimental design used to record Ca^2+^ signals from glutamatergic neuronal populations in the anterior cingulate cortex (ACC) while delivering optogenetic activation to parvalbumin (PV)-expressing neurons. (B–C) Optogenetic activation of ACC PV-expressing neurons inhibits the basal activity of glutamatergic neurons. (B) Heatmap of calcium activity in the ACC of all the PV-Cre mouse recorded following optic stimulation. (C) Average ﬁber photometry response trace of ACC glutamatergic neurons elicited by optic stimulation of PV-expressing neurons (n = 4 mice). Vertical dotted line represents light stimulation (580 nm, 20 Hz, 3 s). Shaded areas in the right panel represent ± standard error of the mean. (D–F) Optogenetic inactivation of ACC PV-expressing neurons enhances the basal activity of glutamatergic neurons. (G–I) Optogenetic activation of ACC somatostatin (SST)-expressing neurons inhibits the basal activity of glutamatergic neurons. (J–L) Optogenetic inactivation of ACC SST-expressing neurons inhibits the basal activity of glutamatergic neurons. The conventions of (D–F), (G–I), and (J–L) are the same as those in (A–C).

**
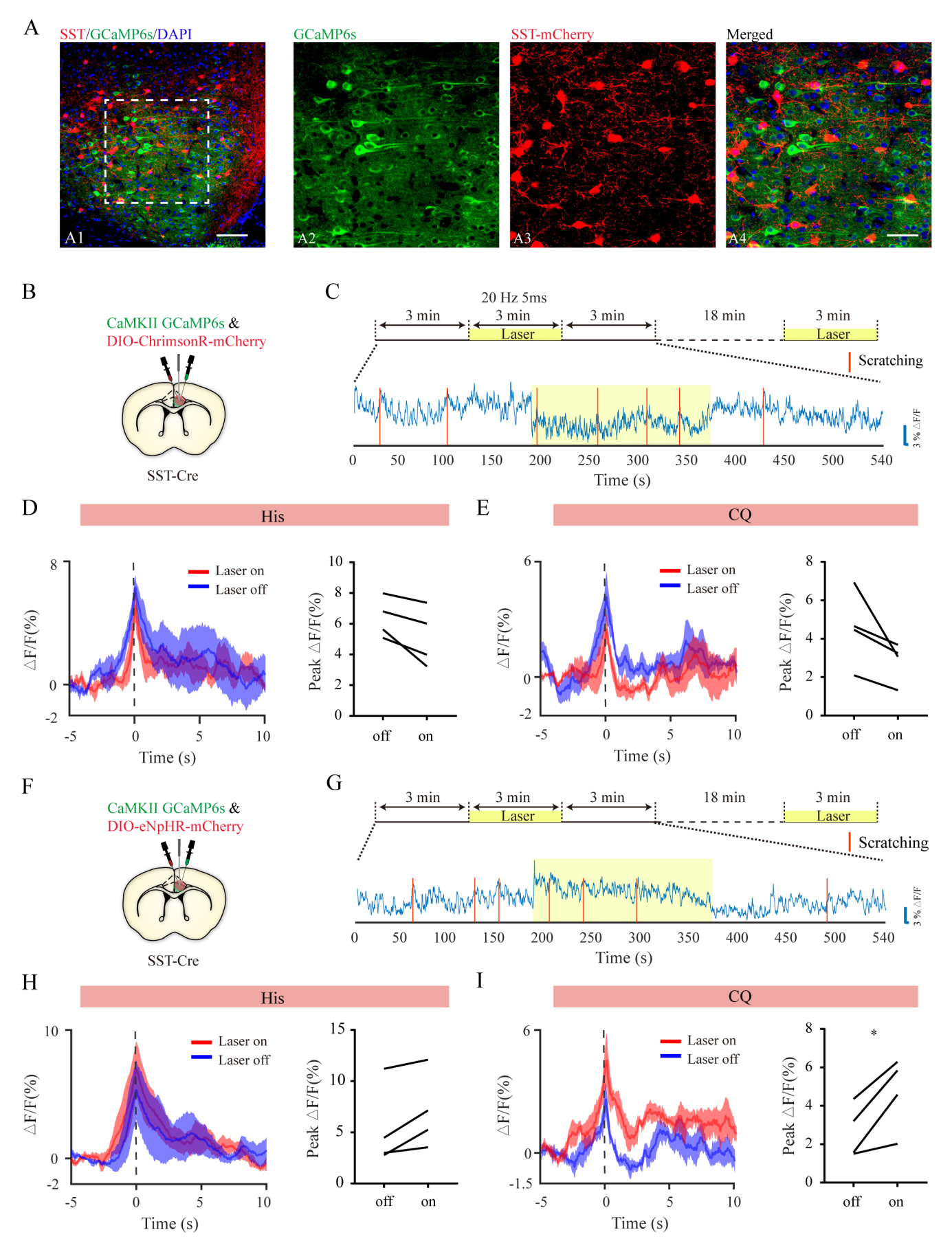
**

**Figure S6.** Effects of optogenetic manipulation of ACC SST-expressing neurons on the activity of glutamatergic neurons during acute itch. (A) Representative photograph showing CaMKII-GCaMP6s labeled glutamatergic neurons (green) and DIO-ChrimsonR-mCherry-labeled somatostatin (SST) neurons (red) in SST-Cre mice. Scale bars: 100 μm (A1) and 50 μm (A2–4). (B) Schematic illustration of the virus injection. (C) A sample trace of calcium ﬂuorescence changes in anterior cingulate cortex (ACC) glutamatergic neurons related to scratching induced by histamine stimuli during light-on and light-off periods. (D) The effect of optical activation of SST-expressing neurons on the activity of glutamatergic neurons in the ACC during histamine-induced scratching. Left panel: Average traces of calcium ﬂuorescence responses in ACC glutamatergic neurons related to histamine-induced acute scratching upon optical stimulation, with shaded areas indicating the standard error of the mean. The red and black lines denote the traces during the light-on and light-off periods, respectively. The vertical dotted line indicates the scratching bouts. Right panel: Average peak ΔF/F of GcaMP6s fluorescence in the light-on and light-off periods under histamine stimulation. N = 4 mice. Paired *t*-test. (E) The effect of optical activation of SST-expressing neurons on glutamatergic neuron activity in the ACC during CQ-induced scratching. The conventions are the same as those in (D). Paired *t*-test. (F–I) Effect of optical inhibition of SST-expressing neurons on the activity of glutamatergic neurons in the ACC during histamine- and CQ-induced scratching. The conventions are the same as those in (E–G). n = 4 mice. Paired *t*-test.


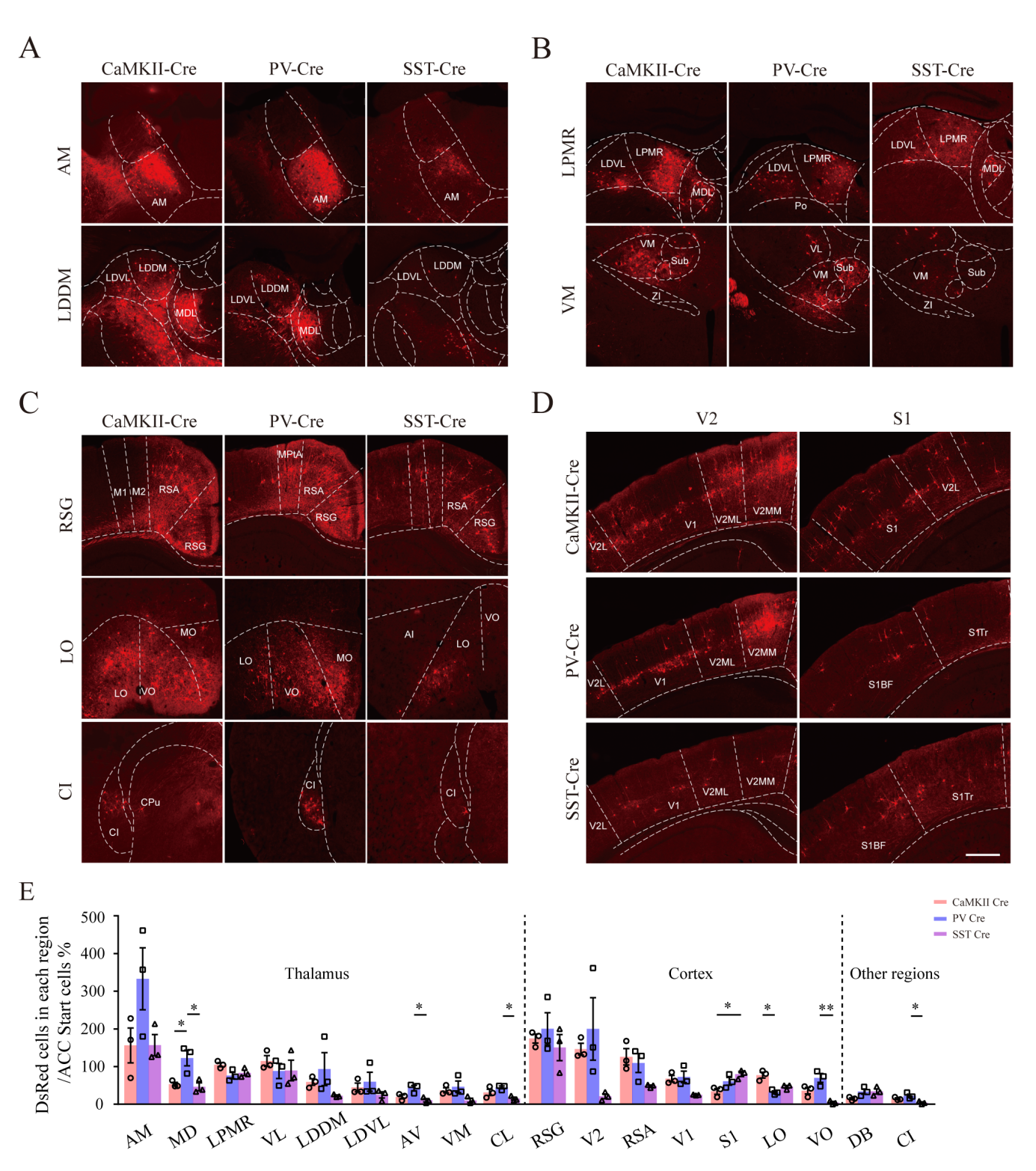


**Figure S7.** Whole-brain monosynaptic afferent inputs to anterior cingulate cortex glutamatergic, PV-expressing, and SST-expressing neurons in mice. (A–D) Representative images indicating input neurons in the ipsilateral thalamic and cortical areas of CaMKII-Cre, parvalbumin (PV)-Cre, and somatostatin (SST)-Cre mice. Scale bars: 1 mm. (E) Normalized distributions of rabies-labeled input neurons across different brain areas ipsilateral to the injection site in CaMKII-Cre, PV-Cre, and SST-Cre mice. Kruskal–Wallis H test or one-way ANOVA followed by Bonferroni test. AM, anteromedial thalamic nucleus; AV, anteroventral thalamic nucleus; Cl, claustrum; DB, nucleus of the diagonal band; LDDM, laterodorsal thalamic nucleus, dorsomedial part; LDVL, laterodorsal thalamic nucleus, ventrolateral part; LO, lateral orbital cortex; LPMR, lateral posterior thalamic nucleus; MD, mediodorsal thalamic nucleus; RSA, retrosplenial agranular cortex; RSG, retrosplenial granular cortex; S1, primary somatosensory cortex; V1, primary visual cortex; V2, secondary visual cortex; VM, ventromedial thalamic nucleus; VO, ventral orbital cortex.


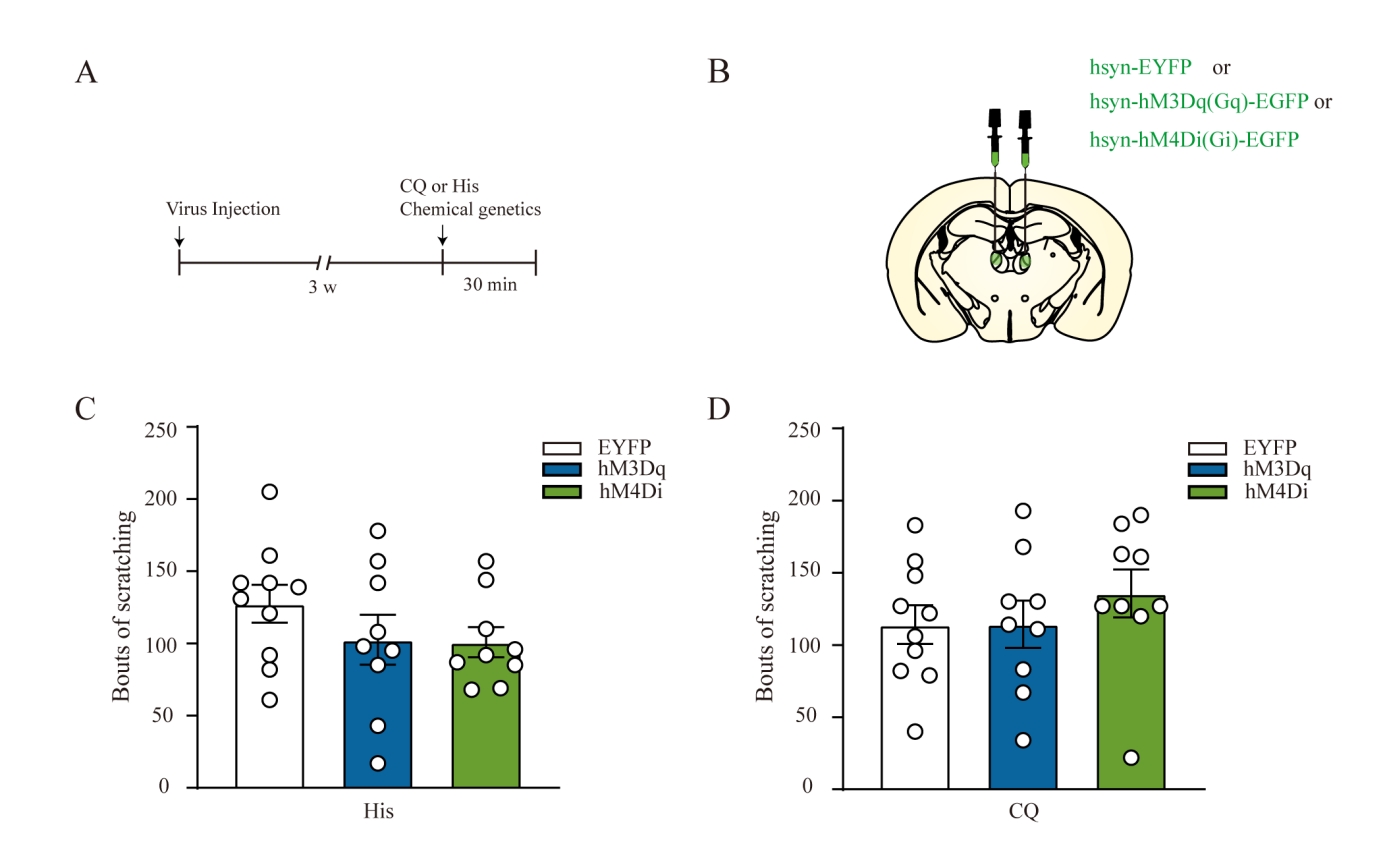


**Figure S8.** Effects of chemogenetic manipulation of MD neurons on acute itch-induced scratching behavior. (A) Timeline of experimental design. (B) Schematic illustration of virus injection for chemogenetic manipulation of bilateral MD neurons. (C-D) Chemogenetic activation or inactivation of MD neurons did not affect scratching behavior induced by histamine (C) and CQ (D). N = 10 mice in the EYFP group and 9 in the hM4Di and hM3Dq groups. One-way ANOVA test.
